# Bayesian Sampling with Approximate Transport Geometry via Residual-Slice Correction

**DOI:** 10.3390/e28080899

**Published:** 2026-08-10

**Authors:** Yuanzheng Zhu, Qiao Hu

**Affiliations:** 1 School of Statistics and Data Science, Southwestern University of Finance and Economics, Chengdu 611130, China; zhuyz@swufe.edu.cn; 2 Department of Economics, The Chinese University of Hong Kong, Hong Kong, China

**Keywords:** Bayesian sampling, Markov chain Monte Carlo, slice sampling, transport preconditioning, residual-slice correction, mixing-loss decomposition, 62F15, 65C05, 65C40, 60J22

## Abstract

Approximate transport maps can facilitate exploration of a Bayesian target distribution, but the resulting samples generally do not follow that distribution. To address this problem, we develop residual-slice correction, a sampling framework that combines slice sampling with an approximate transport map held fixed during sampling. Each iteration uses a slice variable to represent the residual left by the map and updates the state while preserving the conditional distribution on the resulting feasible set. To assess sampling efficiency, we derive a lower bound on the corrected chain’s Dirichlet-form gap using a reference Markov kernel. The bound separates movement within each feasible set, the transport–reference comparison, and reference mixing, while projected diagnostics examine the first two factors. Numerical experiments show that residual-slice correction recovers summaries and shape diagnostics distorted by approximate transport; they also show that the choice of Markov update within each feasible set substantially affects mixing efficiency, and that the corrected chains have lower serial dependence after normalizing-flow training. Overall, the framework retains the geometric benefits of approximate transport while preserving the target distribution.

## 1. Introduction

Bayesian sampling is used to estimate posterior summaries such as means and standard deviations, and to assess distributional shape. Markov chain Monte Carlo (MCMC) methods support this task by constructing chains whose invariant distribution is the target distribution [1,2,3,4,5]. An effective MCMC method must preserve the target distribution and explore its geometry efficiently enough for reliable posterior inference. In practice, however, target geometry often makes efficient exploration difficult. Random-walk and Langevin methods can be slowed by anisotropy and scaling [6,7,8]. Hamiltonian and Riemannian methods use gradient or metric information, but their performance depends on reliable tuning [9,10,11,12]. Directional methods such as hit-and-run exploit convex geometry [13]. These examples point to the same statistical issue: efficient Bayesian sampling depends on how well Markov moves are matched to the target geometry.

Transport maps offer a direct way to improve MCMC geometry. If applying such a map produced target samples, sampling would be essentially direct [14,15]. In practice, the maps available for computation are approximate transport maps constructed from finite-dimensional approximations, Laplace approximations, variational objectives, independent preliminary samples, or flow objectives [16,17,18,19,20,21]. These maps can reduce anisotropy, whiten local curvature, and improve proposal geometry [15,16,21]. However, samples generated directly by an approximate map generally do not follow the target distribution, which can bias uncertainty summaries. This motivates the central problem addressed in this paper: how to use approximate transport maps to improve Markov-chain movement while preserving the target distribution.

The approximate map is constructed before sampling and serves as a fixed preconditioner. In transformed coordinates, a tractable base factor retains useful geometry, while a residual factor records the remaining discrepancy. To correct this residual, we use slice sampling [22,23]. A slice variable converts the positive residual density factor into a level-set constraint. The corrected update then moves among states satisfying this residual constraint. Integrating out the slice variable recovers the target distribution in transformed coordinates. Mapping the resulting chain back gives samples from the target distribution. We call this update residual-slice correction.

The resulting framework separates geometry, correction, and movement. Approximate transport supplies geometric information for Markov moves [15,16], while the slice variable accounts for the residual density factor. The conditional update controls movement on the residual feasible sets. This division is closely related to hybrid slice methods [24], where ideal draws from slice conditional distributions are compared with practical conditional Markov kernels. Normalizing-flow MCMC methods use learned maps to construct global proposals or reparameterize the target [21,25], while transport elliptical slice sampling (TESS) combines a learned map with elliptical slice updates [26,27]. Our framework instead fixes the map before sampling and corrects the remaining discrepancy through slice augmentation and an invariant conditional update.

Within this framework, we establish target preservation through residual-slice augmentation and invariant conditional updates. For efficiency, a product-form lower bound on the Dirichlet-form gap separates within-slice movement, the transport–reference comparison, and reference mixing; projected diagnostics based on selected state summaries examine the first two factors. Numerical experiments show that the correction recovers posterior summaries and distributional shape distorted by approximate transport, while transport quality and the conditional update affect efficiency.

The rest of this paper is organized as follows: Section 2 presents a one-dimensional example, the general algorithm, and three implementable within-slice updates. Section 3 establishes target invariance and develops the mixing-loss diagnostics. Section 4 reports the computational results, followed by the discussion and conclusions in Section 5 and Section 6.

Notation: Let π denote the target distribution and ρ an unnormalized target density. We denote the fixed approximate map by *T*, the transformed variable by $z\in Z\subseteq R^{d}$, and the original variable by $x=T\left(z\right)\in X\subseteq R^{d}$. We write $\tilde{\pi }$ for the target distribution in transformed coordinates, so $z∼\tilde{\pi }$ if and only if T(z)∼π, and denote its unnormalized density by $\tilde{\rho }$. We use $b_{T}$ for the tractable base factor and $\omega_{T}=\tilde{\rho }/b_{T}$ for the residual factor. For a slice level u>0, let $S_{T,u}=\left\{z\in Z:\omega_{T}\left(z\right)>u\right\}$ be the residual feasible set. We denote the residual conditional distribution on $S_{T,u}$ by $\nu_{T,u}$, an invariant kernel for it by $K_{T,u}$, and the number of conditional steps by *m*. For vectors, $∥\cdot ∥$ denotes the Euclidean norm, |·| denotes the absolute value, and 1{·} is an indicator. For a probability measure ϖ, $L_{0}^{2}\left(\varpi \right)$ denotes centered square-integrable functions. If a reversible Markov kernel *P* has invariant distribution ϖ, then ${\mathrm{gap}}_{D}\left(P\right)$ denotes the Dirichlet-form gap. For positive definite matrices, κ denotes the condition number, the ratio of largest to smallest eigenvalue.

## 2. Residual-Slice Correction with a Fixed Transport Preconditioner

This section develops the residual-slice correction framework by first using a fixed approximate map to express the target in transformed coordinates and isolate its residual factor. It then introduces the slice correction, states the implementable update, and describes three within-slice implementations.

### 2.1. Fixed Transport Preconditioning and Transformed Target

Let π be the target distribution on the original space $X\subseteq R^{d}$, and let $Z\subseteq R^{d}$ be the transformed space. Let T:Z→X be the fixed approximate transport map used as a preconditioner. Assume that *T* is a measurable bijection with measurable inverse. Define $\tilde{\pi }$ as the distribution of $z=T^{-1}\left(x\right)$ when x∼π. Equivalently, $z∼\tilde{\pi }$ if and only if T(z)∼π. When X and Z are open, *T* is a $C^{1}$ diffeomorphism, and π admits an unnormalized density ρ, the corresponding unnormalized density in *z* is

$$\tilde{\rho }\left(z\right)=\rho \left(T\left(z\right)\right)\left|\det\nabla T(z)\right|,\;z\in Z.$$

In these coordinates, the target can be easier to explore. For example, if a target is approximately centered at μ with covariance Σ, an affine map T(z)=μ+Lz with $LL^{⊤}\approx \Sigma$ makes the transformed distribution closer to isotropic. A nonlinear map can play the same role for curved targets when it accounts for part of the target curvature. The map *T* therefore preconditions the Markov moves, while a separate correction preserves the target distribution.

For *z* drawn from a chosen simple distribution, T(z) is a target sample exactly when T(z)∼π. In transformed coordinates, choose a positive, tractable base factor $b_{T}$ on Z, which supplies the base weight used to construct within-slice proposals. Define the residual factor $\omega_{T}\left(z\right)=\tilde{\rho }\left(z\right)/b_{T}\left(z\right)$, so that $\tilde{\rho }\left(z\right)=b_{T}\left(z\right)\omega_{T}\left(z\right)$. If $b_{T}$ is proportional to $\tilde{\rho }$, then $\omega_{T}$ is constant and no density discrepancy remains. When $\omega_{T}$ is non-constant, $b_{T}$ describes the geometry retained from the approximate map, and $\omega_{T}$ gives the correction applied by the Markov chain. If T(z)∼π for *z* drawn from that simple distribution and $b_{T}$ is proportional to its density, then $\omega_{T}$ is constant and no residual correction is needed.

**A One-Dimensional Illustration:** Consider the nonlinear target with unnormalized density

$$\rho \left(x\right)=\frac{\exp\left\{-{\mathrm{arsinh}}^{2}\left(x\right)/2\right\}}{\sqrt{1+x^{2}}},\;x\in R.$$

If z∼N(0,1), then $T_{⋆}\left(z\right)=\sinh z$ follows the target distribution. We use the approximate map T(z)=sinh(θ+sz), where θ∈R and s>1. It gives $\mathrm{arsinh}\left\{T\left(z\right)\right\}∼N\left(\theta ,s^{2}\right)$, whereas the target requires N(0,1). The map turns the non-Gaussian target into a Gaussian density in *z*, leaving a location and scale mismatch. The transformed target has unnormalized density

$$\tilde{\rho }\left(z\right)=s\exp\left\{-\frac{{\left(\theta +sz\right)}^{2}}{2}\right\}.$$

Taking $b_{T}\left(z\right)=\exp\left(-z^{2}/2\right)$ as the Gaussian base gives the residual factor

$$\omega_{T}\left(z\right)=\frac{\tilde{\rho }\left(z\right)}{b_{T}\left(z\right)}=s\exp\left[-\frac{{\left(\theta +sz\right)}^{2}-z^{2}}{2}\right].$$

For s>1, this residual factor has maximum

$$\omega_{T}^{\max}=s\exp\left\{\frac{\theta^{2}}{2(s^{2}-1)}\right\},$$

and for $0<u<\omega_{T}^{\max}$, the residual feasible set is the interval

$$S_{T,u}=\left\{z:|z+\frac{\theta s}{s^{2}-1}|<\sqrt{\frac{2}{s^{2}-1}\log\left(\frac{\omega_{T}^{\max}}{u}\right)}\right\},$$

with conditional distribution

$$\nu_{T,u}\left(dz\right)\propto \exp\left(-z^{2}/2\right)1\left\{z\in S_{T,u}\right\}dz.$$

Thus, the conditional distribution is a standard Gaussian restricted to $S_{T,u}$. The three parts of the method now have direct roles: *T* removes the nonlinear shape, $\omega_{T}$ records the remaining location and scale error, and *u* selects an interval for the conditional update. Any kernel preserving the truncated-Gaussian conditional can be used. More conditional steps can improve movement within the interval but require more computation. Overall, the approximate map simplifies the target geometry, while residual-slice correction accounts for the remaining error and preserves the target distribution.

### 2.2. Residual-Slice Correction

The residual factor satisfies the classical slice identity [22,23]:

$$\omega_{T}\left(z\right)=\int_{0}^{\infty }1\left\{0<u<\omega_{T}\left(z\right)\right\}\;du.$$

The resulting joint density is proportional to $b_{T}\left(z\right)1\left\{0<u<\omega_{T}\left(z\right)\right\}$. Marginalizing over *u* recovers $b_{T}\left(z\right)\omega_{T}\left(z\right)=\tilde{\rho }\left(z\right)$, while conditioning on *u* restricts the update to the set where $\omega_{T}$ exceeds the sampled level and weights that set by $b_{T}$. Define the corresponding level-set function by

$$l_{T}\left(u\right)=\int_{Z}b_{T}\left(z\right)1\left\{\omega_{T}\left(z\right)>u\right\}\;dz.$$

For each relevant slice level *u*, the base factor restricted to the feasible set must have a finite, positive normalizing constant. The precise conditions are given in Definition 1.

**Definition 1** 
(Admissible factorization)**.** *The pair $\left(b_{T},\omega_{T}\right)$ is admissible for $\tilde{\rho }=b_{T}\omega_{T}$ on Z if $b_{T}$ is measurable and positive, $\omega_{T}$ is measurable and nonnegative, and*

$$0<\int_{Z}b_{T}\left(z\right)\omega_{T}\left(z\right)dz<\infty ,\;0<\omega_{T}\left(z\right)<\infty \;\tilde{\pi }\text{-}a.s.$$

*and*

$$0<l_{T}\left(u\right)<\infty$$

*for every u>0 such that $\tilde{\pi }\left\{z:\omega_{T}\left(z\right)>u\right\}>0$.*

With admissibility in place, the ideal residual-slice update is well defined. Given *z*, it draws $u∼\mathrm{Unif}(0,\omega_{T}\left(z\right))$ and then draws the next state from the probability measure on Z proportional to $b_{T}\left(y\right)1\left\{\omega_{T}\left(y\right)>u\right\}dy$. This ideal update is reversible with respect to $\tilde{\pi }$ by the slice-augmentation argument [3,22,23]. The practical version replaces the exact conditional draw by a Markov transition that preserves the same conditional distribution.

In the one-dimensional example, *u* selects a finite interval, and the second step preserves the standard-Gaussian distribution restricted to that interval. Thus, $b_{T}$ supplies the base geometry, $\omega_{T}$ supplies the residual correction, and the conditional kernel supplies the movement. We next specify the implementable update.

### 2.3. General Residual-Slice Update

For a level u>0, define the residual feasible set $S_{T,u}=\left\{z\in Z:\omega_{T}\left(z\right)>u\right\}$. When $0<\int_{Z}b_{T}\left(y\right)1\left\{y\in S_{T,u}\right\}dy<\infty$, define the residual conditional distribution by

(1) $$\nu_{T,u}\left(dz\right)=\frac{b_{T}\left(z\right)1\left\{z\in S_{T,u}\right\}dz}{\int_{Z}b_{T}\left(y\right)1\left\{y\in S_{T,u}\right\}dy}.$$

The ideal update draws exactly from $\nu_{T,u}$. The implementable version applies *m* transitions of $K_{T,u}$, which preserves $\nu_{T,u}$, after drawing *u*. Every fixed m≥1 preserves the same marginal target because $K_{T,u}^{m}$ is also invariant for $\nu_{T,u}$. Algorithm 1 states the complete update. The efficiency comparisons in Section 3 also assume reversibility.

**Algorithm 1** Residual-slice correction with a fixed transport preconditioner**Input:** Unnormalized target density ρ, fixed transport preconditioner *T*, base factor $b_{T}$, residual factor $\omega_{T}$, residual-conditional kernels ${\left\{K_{T,u}\right\}}_{u>0}$, initial transformed state $z_{0}$ satisfying $0<b_{T}\left(z_{0}\right)<\infty$ and $0<\omega_{T}\left(z_{0}\right)<\infty$, fixed kernel parameters, number of outer transitions $N_{\mathrm{out}}$ and inner steps *m*.
For $t=0,\ldots ,N_{\mathrm{out}}-1$, take $z_{t}$ as the current state and perform the following update:
(a)Draw $u_{t}∼\mathrm{Unif}\left(0,\omega_{T}\left(z_{t}\right)\right)$.(b)Starting from $z_{t}$, apply *m* steps of the prespecified kernel $K_{T,u_{t}}$, each preserving $\nu_{T,u_{t}}$ in (1).(c)Store the final state as $z_{t+1}$.After the loop, map the stored transformed states once by $x_{t}=T\left(z_{t}\right)$, $t=1,\ldots ,N_{\mathrm{out}}$, to obtain original-space output.

Algorithm 1 separates the correction principle from the proposal used inside a residual feasible set. Target invariance follows from the invariant conditional Markov update, while efficiency depends on the movement of $K_{T,u}$ within $S_{T,u}$. The kernel $K_{T,u}$ may be any prespecified transition that preserves $\nu_{T,u}$. We consider three implementations: random-walk (RW) hybrid slice, gradient-assisted corrected transport-slice (Gradient CTS), and preconditioned Crank–Nicolson corrected transport-slice (pCN CTS). For each implementation, the transport and proposal parameters are fixed before retained sampling and do not depend on the realized slice level *u* or retained-chain history. Their proposals and acceptance rules are described below and summarized in Table 1. Convergence additionally requires the usual irreducibility and aperiodicity conditions for the chosen conditional kernels.

A generic within-slice Metropolis implementation is obtained by correcting a proposal inside each residual feasible set. Suppose that $R_{T,u}\left(z,dy\right)$ is a transformed-space proposal with density $r_{T,u}\left(z,y\right)$ with respect to a common reference measure on the proposed move space. For $z\in S_{T,u}$, propose $y∼R_{T,u}\left(z,\cdot \right)$ and accept it with probability

(2) $$\alpha_{T,u}\left(z,y\right)=\left\{\begin{array}{cc} 1\wedge \frac{b_{T}\left(y\right)r_{T,u}\left(y,z\right)}{b_{T}\left(z\right)r_{T,u}\left(z,y\right)},\; & y\in S_{T,u},\\ 0, & y\notin S_{T,u}, \end{array}\right.$$

with the usual convention that the ratio is used only where the denominator is positive. If the proposal is rejected, the chain remains at *z*. The piecewise rule in (2) enforces slice feasibility, while the Metropolis ratio corrects the proposal to the conditional density in (1). Symmetric proposals on the slice reduce the ratio to $b_{T}\left(y\right)/b_{T}\left(z\right)$, which is computationally simple for base factors with tractable density ratios.

Here, $\xi ∼N(0,I_{d})$; the pCN row uses the Gaussian base $b_{T}\left(z\right)=\exp\left\{-\tau {∥z∥}^{2}\right\}$, with τ>0.

### 2.4. Within-Slice Kernel Choices

The remaining algorithmic choice is the within-slice kernel $K_{T,u}$. All three choices below preserve the residual conditional distribution in (1), but they differ in how they propose a feasible move. As a matched baseline, the RW hybrid slice update serves as the residual-slice comparison. It uses

$$y=z+\sigma \xi ,\;\xi ∼N(0,I_{d}),$$

where σ>0 is fixed. Using the same transport and factorization isolates the effect of random-walk movement within the residual conditional.

A second implementation, Gradient CTS, uses base-factor gradient information while retaining the same feasibility and Metropolis correction. Its proposal is

$$y=z+\frac{\epsilon^{2}}{2}\nabla \log b_{T}\left(z\right)+\epsilon \xi ,\;\xi ∼N\left(0,I_{d}\right),$$

where ϵ>0 is fixed. Equation (2) corrects the proposal to the conditional density proportional to $b_{T}\left(z\right)1\left\{z\in S_{T,u}\right\}$, while the base gradient guides movement within $S_{T,u}$.

For Gaussian base factors, the same correction also admits a reversible non-gradient option. When

$$\log b_{T}\left(z\right)=-\tau {∥z∥}^{2},\;\tau >0,$$

the pCN CTS implementation uses the proposal [28]

$$y=\sqrt{1-\beta^{2}}\;z+\frac{\beta }{\sqrt{2\tau }}\xi ,\;\xi ∼N\left(0,I_{d}\right),\;0<\beta <1.$$

The parameter β controls the pCN proposal scale. This proposal is reversible with respect to the normalized Gaussian base associated with $b_{T}$. Therefore, after rejection outside $S_{T,u}$, it is reversible with respect to the residual conditional distribution in (1).

For the pCN implementation of the one-dimensional example, $b_{T}\left(z\right)=\exp\left(-z^{2}/2\right)$, so τ=1/2. Fix *T*, β, *m*, and $z_{0}$ before the retained chain begins. Given $z_{t}$, draw $u_{t}∼\mathrm{Unif}\left(0,\omega_{T}\left(z_{t}\right)\right)$ and form the interval $S_{T,u_{t}}$. Starting at $z_{t}$, make *m* proposals $y=\sqrt{1-\beta^{2}}\;z+\beta \xi$, where ξ∼N(0,1). Accept a proposal in $S_{T,u_{t}}$ and reject a proposal outside it. The state after these *m* proposals is $z_{t+1}$. After all transitions, apply *T* to the stored states. Section 3 next separates target preservation from the efficiency effects of transport quality and within-slice movement.

## 3. Target Invariance, Mixing-Loss Decomposition, and Diagnostics

This section establishes target preservation and then analyzes efficiency. The resulting bound separates within-slice movement, transport–reference comparison, and reference mixing, with projected diagnostics based on selected state summaries for the first two factors.

### 3.1. Target Invariance of Residual-Slice Correction

The first result formalizes the target-preservation role of the slice correction. Let ${\tilde{P}}_{T}^{\mathrm{hyb},m}$ be the transformed-space kernel that samples a slice level from the current point and then applies *m* steps of $K_{T,u}$. Its original-space conjugate is

$$P_{T}^{\mathrm{hyb},m}\left(x,B\right)={\tilde{P}}_{T}^{\mathrm{hyb},m}\left(T^{-1}\left(x\right),T^{-1}\left(B\right)\right).$$

The associated joint slice measure is

$$\eta_{T}\left(dz,du\right)=Z_{T}^{-1}b_{T}\left(z\right)1\left\{0<u<\omega_{T}\left(z\right)\right\}dz\;du,\;Z_{T}=\int_{Z}b_{T}\left(z\right)\omega_{T}\left(z\right)dz,$$

and $\eta_{T}^{\left(u\right)}$ denotes its level marginal distribution. For comparison with an exact draw from the residual conditional distribution, let ${\tilde{P}}_{T}^{\mathrm{ideal}}$ denote the transformed-space kernel that samples exactly from (1). For any measurable original-space set B⊆X, with $\tilde{B}=T^{-1}\left(B\right)$, its original-space version is

$$P_{T}^{\mathrm{ideal}}\left(x,B\right)={\tilde{P}}_{T}^{\mathrm{ideal}}\left(T^{-1}\left(x\right),\tilde{B}\right).$$

The correction is visible directly from this joint density: integrating out *u* gives

$$\int_{0}^{\infty }b_{T}\left(z\right)1\left\{0<u<\omega_{T}\left(z\right)\right\}\;du=b_{T}\left(z\right)\omega_{T}\left(z\right)=\tilde{\rho }\left(z\right).$$

Thus, the slice level does not approximate the target; it is an auxiliary representation whose marginal is exactly the target distribution in transformed coordinates.

Assumption 1 combines the admissibility condition in Definition 1 with invariance of the implemented within-slice kernels.

**Assumption 1.** 
*For the fixed map T, the pair $\left(b_{T},\omega_{T}\right)$ is admissible on Z in the sense of Definition 1. Moreover, $K_{T,u}$ is invariant with respect to $\nu_{T,u}$ for $\eta_{T}^{\left(u\right)}$-almost every u.*

This is the standard conditional-invariance requirement for auxiliary-variable and hybrid slice samplers: an exact conditional draw may be replaced by a transition that preserves the conditional distribution [3,24].

**Theorem 1.** 
*Under Assumption 1, ${\tilde{P}}_{T}^{\mathrm{hyb},m}$ is invariant with respect to $\tilde{\pi }$ for every m≥1, and $P_{T}^{\mathrm{hyb},m}$ is invariant with respect to π. In addition, if $K_{T,u}$ is reversible with respect to $\nu_{T,u}$ for $\eta_{T}^{\left(u\right)}$-almost every u, then the two hybrid kernels are reversible with respect to their corresponding target distributions.*

The theorem combines classical slice marginalization with invariant conditional updates and transfers the result between transformed and original spaces through the fixed map *T* [3,22,23].

### 3.2. Product-Form Mixing-Loss Bound

To quantify this efficiency question, the analysis compares the implemented update with an ideal transported slice update. This comparison measures the loss incurred when an ideal draw from the residual conditional distribution is replaced by *m* corrected within-slice steps. For a reversible kernel $K_{T,u}^{m}$, its Dirichlet form on $L^{2}\left(\nu_{T,u}\right)$ is the expected squared change

$$E_{K_{T,u}^{m}}^{\left(u\right)}\left(f,f\right)=\frac{1}{2}\int {\left\{f\left(z\right)-f\left(y\right)\right\}}^{2}\nu_{T,u}\left(dz\right)K_{T,u}^{m}\left(z,dy\right).$$

It is zero when the selected observable does not move and increases as the *m*-step conditional update produces larger changes. The within-slice movement factor is the induced comparison constant

(3) $$c_{\mathrm{slice}}=\underset{\begin{array}{c} f\in L_{0}^{2}\left(\tilde{\pi }\right)\\ \int {\mathrm{Var}}_{\nu_{T,u}}\left(f\right)\eta_{T}^{\left(u\right)}\left(du\right)>0 \end{array}}{\inf}\frac{\int E_{K_{T,u}^{m}}^{\left(u\right)}\left(f,f\right)\eta_{T}^{\left(u\right)}\left(du\right)}{\int {\mathrm{Var}}_{\nu_{T,u}}\left(f\right)\eta_{T}^{\left(u\right)}\left(du\right)}.$$

By definition, for every $f\in L_{0}^{2}\left(\tilde{\pi }\right)$,

(4) $$\int E_{K_{T,u}^{m}}^{\left(u\right)}\left(f,f\right)\eta_{T}^{\left(u\right)}\left(du\right)\geq c_{\mathrm{slice}}\int {\mathrm{Var}}_{\nu_{T,u}}\left(f\right)\eta_{T}^{\left(u\right)}\left(du\right).$$

This transported, level-averaged comparison is the analog of the conditional-mixing comparison used in hybrid slice-sampling analyses, where the implemented transition on each slice is compared with the ideal draw from the slice conditional distribution [24]. Smaller values of $c_{\mathrm{slice}}$ correspond to less movement within slices. If the denominator in (3) is zero for a function, that function imposes no restriction on the comparison. For a reversible kernel *P* with invariant distribution ϖ, write

$${\mathrm{gap}}_{D}\left(P\right)=\underset{f\in L_{0}^{2}\left(\varpi \right),\;f\neq 0}{\inf}\frac{E_{P}\left(f,f\right)}{{\mathrm{Var}}_{\varpi }\left(f\right)}.$$

The next proposition records the resulting hybrid-to-ideal transfer in the transported residual-slice setting.

**Proposition 1.** 
*Let Assumption 1 hold, and suppose that $K_{T,u}$ is reversible with respect to $\nu_{T,u}$ for $\eta_{T}^{\left(u\right)}$-almost every u. Then, the transformed hybrid kernel satisfies*

$${\mathrm{gap}}_{D}\left({\tilde{P}}_{T}^{\mathrm{hyb},m}\right)\geq c_{\mathrm{slice}}\;{\mathrm{gap}}_{D}\left({\tilde{P}}_{T}^{\mathrm{ideal}}\right).$$

*The original-space conjugate then satisfies*

$${\mathrm{gap}}_{D}\left(P_{T}^{\mathrm{hyb},m}\right)\geq c_{\mathrm{slice}}\;{\mathrm{gap}}_{D}\left(P_{T}^{\mathrm{ideal}}\right).$$

Because (4) averages over slice levels, it requires sufficient average movement across levels [24]. For positive reversible kernels, or lazy versions of reversible ones, the same comparison can be read in terms of the usual absolute $L^{2}$ spectral gap, with the standard laziness factor absorbed into $c_{\mathrm{slice}}$.

Ideal residual-conditional draws still reflect the transformed geometry, so we compare their Dirichlet-form movement with a reversible $\tilde{\pi }$-invariant reference kernel $Q_{T}$, whose gap may be known or estimated [29]. Define the transport–reference comparison factor and reference mixing scale by

(5) $$a_{T}=\underset{\begin{array}{c} f\in L_{0}^{2}\left(\tilde{\pi }\right)\\ E_{Q_{T}}\left(f,f\right)>0 \end{array}}{\inf}\frac{E_{{\tilde{P}}_{T}^{\mathrm{ideal}}}\left(f,f\right)}{E_{Q_{T}}\left(f,f\right)},\;\gamma_{\mathrm{ref}}={\mathrm{gap}}_{D}\left(Q_{T}\right).$$

This definition gives the transport–reference comparison

(6) $$E_{{\tilde{P}}_{T}^{\mathrm{ideal}}}\left(f,f\right)\geq a_{T}\;E_{Q_{T}}\left(f,f\right),\;f\in L_{0}^{2}\left(\tilde{\pi }\right).$$

The factor $a_{T}$ depends on the base–residual split and reference construction, so comparisons across maps require a consistent reference family. Combining this comparison with Proposition 1 gives the product-form bound.

**Theorem 2.** 
*Under the conditions of Proposition 1, let $Q_{T}$ be a reversible $\tilde{\pi }$-invariant reference kernel. Then,*

(7) $${\mathrm{gap}}_{D}\left(P_{T}^{\mathrm{hyb},m}\right)\geq c_{\mathrm{slice}}a_{T}\gamma_{\mathrm{ref}}.$$

Theorem 2 is the main mixing-loss decomposition. It combines the hybrid-to-ideal comparison in Proposition 1 with the transport–reference comparison (6). The factor $c_{\mathrm{slice}}$ measures within-slice movement, $a_{T}$ gives the transport–reference comparison, and $\gamma_{\mathrm{ref}}$ supplies the reference mixing scale. When all three factors are positive, their product gives an explicit bound; the individual factors show where efficiency is lost.

### 3.3. Diagnostics for the Comparison Factors

The population factors in (3) and (5) are global infima over $L_{0}^{2}\left(\tilde{\pi }\right)$. To examine their behavior on selected state summaries, let $\psi ={\left(\psi_{1},\ldots ,\psi_{r}\right)}^{⊤}$ be centered functions in $L_{0}^{2}\left(\tilde{\pi }\right)$, and write $V_{\psi }=\mathrm{span}\left\{\psi_{1},\ldots ,\psi_{r}\right\}$. For $f,g\in L^{2}\left(\nu_{T,u}\right)$, define the bilinear Dirichlet form by

$$E_{K_{T,u}^{m}}^{\left(u\right)}\left(f,g\right)=\frac{1}{2}\int \left\{f\left(z\right)-f\left(y\right)\right\}\left\{g\left(z\right)-g\left(y\right)\right\}\nu_{T,u}\left(dz\right)K_{T,u}^{m}\left(z,dy\right).$$

Define the projected ideal and corrected movement matrices by

(8) $$\begin{array}{c} G_{\mathrm{hyb}}\left(i,j\right)=\int E_{K_{T,u}^{m}}^{\left(u\right)}\left(\psi_{i},\psi_{j}\right)\eta_{T}^{\left(u\right)}\left(du\right),\;G_{\mathrm{ideal}}\left(i,j\right)=\int {\mathrm{Cov}}_{\nu_{T,u}}\left(\psi_{i},\psi_{j}\right)\eta_{T}^{\left(u\right)}\left(du\right). \end{array}$$

These matrices describe the chosen observable span. A small projected eigenvalue reveals poor movement in that span; global behavior requires separate analysis. When the matrix $G_{\mathrm{ideal}}$ in (8) is positive definite on the span of ψ,

(9) $$c_{\mathrm{slice}}^{\left(r\right)}=\lambda_{\min}\left(G_{\mathrm{ideal}}^{-1/2}G_{\mathrm{hyb}}G_{\mathrm{ideal}}^{-1/2}\right).$$

For a reference kernel $Q_{T}$, set $G_{Q}\left(i,j\right)=E_{Q_{T}}\left(\psi_{i},\psi_{j}\right)$. When $G_{Q}$ is positive definite on the span of ψ, define the projected transport diagnostic by

$$a_{T}^{\left(r\right)}=\lambda_{\min}\left(G_{Q}^{-1/2}G_{\mathrm{ideal}}G_{Q}^{-1/2}\right).$$

These are projected population factors, not finite-sample estimates. The next theorem relates them to the population comparisons in (4) and (6).

**Theorem 3.** 
*If $G_{\mathrm{ideal}}$ is positive definite on $V_{\psi }$, then $c_{\mathrm{slice}}^{\left(r\right)}$ in *(9)* is the sharp constant for the integrated slice comparison *(4)* restricted to functions in $V_{\psi }$. Similarly, if $G_{Q}$ is positive definite on $V_{\psi }$, then $a_{T}^{\left(r\right)}$ is the sharp constant for the transport comparison *(6)* restricted to functions in $V_{\psi }$.*

Theorem 3 gives projected factors for state summaries such as components, radii, log densities, predictive summaries, or principal components. The following algorithm estimates these factors from stationary state–slice pairs and paired moves.

Algorithm 2 estimates the projected factors in Theorem 3; hats distinguish the finite-sample matrices and ratios from their population counterparts. If $z_{l}∼\tilde{\pi }$, then drawing $u_{l}∼\mathrm{Unif}\left(0,\omega_{T}\left(z_{l}\right)\right)$ gives a stationary pair; other starting states yield local movement summaries. The ridge λ provides numerical regularization, and δ can incorporate a valid finite-sample error allowance. Low projected movement indicates slow conditional exploration.

**Algorithm 2** Estimating the projected comparison factors**Input:** Fixed transport preconditioner *T*, base factor $b_{T}$, residual factor $\omega_{T}$, within-slice transition $K_{T,u}^{m}$, optional reference kernel $Q_{T}$, centered observables ψ, a collection ${\left\{\left(z_{l},u_{l}\right)\right\}}_{l=1}^{L}$ of stationary joint pairs from $\eta_{T}$, ridge λ≥0, and error allowance δ≥0.
For each stationary pair $\left(z_{l},u_{l}\right)$, draw $y_{l}^{⋆}∼\nu_{T,u_{l}}$ independently of $z_{l}$ conditional on $u_{l}$, and set$${\hat{G}}_{\mathrm{ideal}}=\frac{1}{2L}\sum_{l=1}^{L}\left\{\psi \left(z_{l}\right)-\psi \left(y_{l}^{⋆}\right)\right\}{\left\{\psi \left(z_{l}\right)-\psi \left(y_{l}^{⋆}\right)\right\}}^{⊤}.$$Conditional on $u_{l}$, the factor 1/2 makes this paired-difference outer product an unbiased estimate of the conditional covariance matrix.Estimate the corrected movement matrix from paired before–after moves. For each joint pair $\left(z_{l},u_{l}\right)$, let $y_{l}$ be the state obtained after one application of $K_{T,u_{l}}^{m}$. Set$${\hat{G}}_{\mathrm{hyb}}=\frac{1}{2L}\sum_{l=1}^{L}\left\{\psi \left(z_{l}\right)-\psi \left(y_{l}\right)\right\}{\left\{\psi \left(z_{l}\right)-\psi \left(y_{l}\right)\right\}}^{⊤}.$$If $Q_{T}$ is used, independently draw $q_{l}∼Q_{T}\left(z_{l},\cdot \right)$ from each of the same starting states and set$${\hat{G}}_{Q}=\frac{1}{2L}\sum_{l=1}^{L}\left\{\psi \left(z_{l}\right)-\psi \left(q_{l}\right)\right\}{\left\{\psi \left(z_{l}\right)-\psi \left(q_{l}\right)\right\}}^{⊤}.$$Choose λ so that ${\hat{G}}_{\mathrm{ideal}}+\lambda I≻0$ and, if $Q_{T}$ is used, ${\hat{G}}_{Q}+\lambda I≻0$. Then, compute the projected within-slice diagnostic and, when applicable, the projected transport diagnostic:$${\hat{c}}_{\mathrm{slice}}^{\left(r\right)}\left(\lambda ,\delta \right)=\max\left[0,\;\lambda_{\min}\left\{{\left({\hat{G}}_{\mathrm{ideal}}+\lambda I\right)}^{-1/2}\left({\hat{G}}_{\mathrm{hyb}}-\delta I\right){\left({\hat{G}}_{\mathrm{ideal}}+\lambda I\right)}^{-1/2}\right\}\right].$$When $Q_{T}$ is used, compute the projected transport diagnostic directly as$${\hat{a}}_{T}^{\left(r\right)}\left(\lambda ,\delta \right)=\max\left[0,\;\lambda_{\min}\left\{{\left({\hat{G}}_{Q}+\lambda I\right)}^{-1/2}\left({\hat{G}}_{\mathrm{ideal}}-\delta I\right){\left({\hat{G}}_{Q}+\lambda I\right)}^{-1/2}\right\}\right].$$


### 3.4. Affine Transport Diagnostics

In addition to the projected comparison factors, the affine diagnostics below assess second-order geometry and do not directly estimate $a_{T}$. A Laplace-affine preconditioner centers at a posterior mode and scales by the inverse square root of its Hessian. For comparison, an independent-sample covariance preconditioner centers and scales using the empirical mean and covariance of independent draws; the resulting transformed covariance provides a global whitening diagnostic. The first diagnostic checks local curvature after Laplace-affine preconditioning. It is the local analog of condition-number analyses for gradient-based Markov chains and transport-map preconditioning, where whitening is assessed through the Hessian of the transformed potential [6,8,16]. Write ρ(x)=exp{−U(x)}, let $\hat{x}$ be a local mode, and set $H=\nabla^{2}U\left(\hat{x}\right)≻0$. Define the Laplace-affine transport and transformed potential by

$$T_{L}\left(z\right)=\hat{x}+H^{-1/2}z,\;{\tilde{U}}_{L}\left(z\right)=U\left(T_{L}\left(z\right)\right)-\log\left|\det H^{-1/2}\right|.$$

For R>0, let $E_{R}=\left\{z:∥z∥\leq R\right\}$, and assume that $U\in C^{2}$ on an open set containing $T_{L}\left(E_{R}\right)$. Define

(10) $$\varepsilon_{R}=\underset{z\in E_{R}}{\sup}{∥H^{-1/2}\nabla^{2}U\left(T_{L}\left(z\right)\right)H^{-1/2}-I∥}_{\mathrm{op}}.$$

The radius-wise error in (10) gives a direct local conditioning bound. For a twice-differentiable potential *V*, being α-strongly convex and *L*-smooth on $E_{R}$ means that $\alpha I⪯\nabla^{2}V\left(z\right)⪯LI$ for every $z\in E_{R}$. When $\nabla^{2}{\tilde{U}}_{L}\left(z\right)≻0$ throughout $E_{R}$, define the regional Hessian condition number by

$$\kappa_{R}\left({\tilde{U}}_{L}\right)=\frac{\underset{z\in E_{R}}{\sup}\lambda_{\max}\left\{\nabla^{2}{\tilde{U}}_{L}\left(z\right)\right\}}{\underset{z\in E_{R}}{\inf}\lambda_{\min}\left\{\nabla^{2}{\tilde{U}}_{L}\left(z\right)\right\}}.$$

The following proposition records the deterministic implication for the transformed potential on the chosen finite-radius region.

**Proposition 2.** 
*If $\varepsilon_{R}<1$, then ${\tilde{U}}_{L}$ is $\left(1-\varepsilon_{R}\right)$-strongly convex and $\left(1+\varepsilon_{R}\right)$-smooth on $E_{R}$. Consequently, the Hessian condition number of the transformed potential on $E_{R}$ is at most*

(11) $$\kappa_{R}\left({\tilde{U}}_{L}\right)\leq \frac{1+\varepsilon_{R}}{1-\varepsilon_{R}}.$$

The second diagnostic checks whether independent-sample information has reduced global second-order anisotropy. Let x∼π have mean μ and positive definite covariance Σ. For independent preliminary values $x_{1},\ldots ,x_{N}$, define the explicitly centered estimates

$${\hat{\mu }}_{N}=\frac{1}{N}\sum_{i=1}^{N}x_{i},\;{\hat{\Sigma }}_{N}=\frac{1}{N-1}\sum_{i=1}^{N}\left(x_{i}-{\hat{\mu }}_{N}\right){\left(x_{i}-{\hat{\mu }}_{N}\right)}^{⊤}.$$

Given the estimates ${\hat{\mu }}_{N}$ and ${\hat{\Sigma }}_{N}≻0$, define

$$T_{N}\left(z\right)={\hat{\mu }}_{N}+{\hat{\Sigma }}_{N}^{1/2}z.$$

The covariance-whitening event at tolerance ε∈[0,1) is

(12) $${∥\Sigma^{-1/2}{\hat{\Sigma }}_{N}\Sigma^{-1/2}-I∥}_{\mathrm{op}}\leq \varepsilon .$$

The next proposition translates this event into a condition-number bound for the covariance in transformed coordinates.

**Proposition 3.** 
*On the event *(12)*, the transformed variable $z=T_{N}^{-1}\left(x\right)$ satisfies*

$$\kappa \left\{\mathrm{Cov}\left(z\right)\right\}\leq \frac{1+\varepsilon }{1-\varepsilon }.$$

As ε decreases, the bound approaches one; when ε=0, the transformed covariance is the identity. Proposition 2 concerns local curvature, whereas Proposition 3 concerns global covariance anisotropy. These diagnostics assess second-order conditioning but do not capture angular or tail behavior.

## 4. Computational Results

The experiments examine three aspects of residual-slice correction: recovery of summaries and distributional shape distorted by samples generated from an approximate map, efficiency differences among within-slice updates under the same fixed map, and the effects of transport quality and problem size on corrected sampling. The study covers smooth, non-smooth, nonlinear, and Gaussian targets, together with parameter sensitivity, computational cost, scaling, and a learned transport. Hamiltonian Monte Carlo (HMC), the No-U-Turn sampler (NUTS), the Metropolis-adjusted Langevin algorithm (MALA), random-walk Metropolis (RWM), and analytic references provide trajectory-based and closed-form reference points for the same target distributions.

The output summaries are integrated autocorrelation time (IACT), effective sample size (ESS) per density evaluation (ESS/eval), ESS per second (ESS/sec), acceptance or feasibility rates, and observable-space diagnostic proxies. IACT and ESS/eval are standard efficiency summaries for MCMC output [4,30]. IACT is the maximum initial-positive-sequence estimate over the first two components and squared radius, and ESS is the retained draw count divided by this IACT. ESS/eval and ESS/sec provide evaluation- and time-normalized comparisons; the cost model below accounts for density, gradient, map, Jacobian, and feasibility evaluations. Replicated results are summarized by means; parenthesized values give between-run standard deviations (SDs) where displayed. Proposition A1 verifies the residual-split conditions; the implementation is provided as Supplementary Code S1.

### 4.1. Computational Setup, Tuning, and Cost Model

Each comparison fixes the target, map, and proposal settings before retained sampling. ESS/sec times the executed chain: fixed-parameter methods include burn-in but exclude map construction and scale selection, whereas BlackJAX NUTS includes warm-up, adaptation, and first-call compilation. Because the scopes differ, cross-family ESS/sec is interpreted with the matched comparisons and component-cost breakdown rather than as an end-to-end ranking. The Appendix A (Table A1) lists the fixed residual-slice settings.

Multivariate ESS uses the determinant-ratio batch-means estimator of Vats et al. [30] and is reported when the number of batches exceeds the state dimension. For HMC, the energy Bayesian fraction of missing information (E-BFMI) is the mean squared successive energy difference divided by the energy variance. A transition is classified as divergent when its energy error is non-finite or exceeds $10^{3}$ in absolute value.

Let $C_{\rho },C_{\nabla \rho },C_{T},C_{J}$, and $C_{b}$ denote target-density, density-gradient, map, log-Jacobian and base-factor evaluations, respectively; $C_{b}$ includes the base gradient when required. A joint map–Jacobian call is denoted by $C_{T,J}$. For the Gaussian base, one residual feasibility check costs $C_{T}+C_{J}+C_{\rho }+O\left(d\right)$, or $C_{T,J}+C_{\rho }+O\left(d\right)$ with a joint call. The learned nonlinear map is a real-valued non-volume-preserving (Real NVP) flow. Table 2 separates construction from per-transition cost; *m* is the number of within-slice proposals, while $L_{\mathrm{HMC}}$, $D_{\mathrm{NUTS}}$, and $N_{\mathrm{br}}$ denote the HMC leapfrog count, realized NUTS tree depth, and fixed-flow TESS-style bracket evaluations, respectively.

For a joint map–Jacobian implementation, $C_{T}+C_{J}$ in the residual-slice rows is replaced by $C_{T,J}$. A dense affine map has $O(d^{2})$ evaluation cost, with a cached O(1) log-determinant. In a generalized linear model, $k_{\mathrm{full}}$ Newton steps typically cost $O\{k_{\mathrm{full}}\left(nd^{2}+d^{3}\right)\}$. A directly fitted diagonal approximation costs $O(k_{\mathrm{diag}}nd)$ over $k_{\mathrm{diag}}$ first-order steps and has O(d) evaluation cost. For a Real NVP with $q_{\mathrm{flow}}$ coupling blocks and width *h*, a joint map–Jacobian call costs $O\{q_{\mathrm{flow}}\left(dh+h^{2}\right)\}$. Table 3 reports measured component timings for the d=20,n=900 logistic target.

The full-Laplace fit took 4.8 ms. Gradient CTS, pCN CTS, and RW hybrid slice required 0.602, 0.593, and 0.591 ms per transition, respectively. Here, the target density dominates: the affine map is about 2.2% of a residual evaluation, and the cached Jacobian and Boolean check are each below 0.12%. Separately timed component medians are not additive.

As a trajectory-based reference method, NUTS is included for the smooth logistic target. For the non-smooth lasso and imperfect nonlinear-transport experiments, the comparisons focus on corrected sampling under a fixed transport preconditioner.

### 4.2. Bias from Uncorrected Map Samples and Corrected Recovery

The first comparison measures uncertainty-summary errors in samples generated by the approximate map without correction. It uses a 20-dimensional synthetic logistic-regression target and fixed Laplace-affine maps. The transport-only rows in Table 4 use independent draws from the corresponding Gaussian transport approximation, while the corrected rows use the same fixed preconditioner inside residual-slice correction. Component-wise means and standard deviations are compared with an affine HMC reference chain.

The table lists the maximum and root-mean-square (RMS) standardized mean error,

$$\max_{j}\frac{|{\hat{\mu }}_{j}-\mu_{j}^{\mathrm{ref}}|}{\sigma_{j}^{\mathrm{ref}}},\;{\left\{d^{-1}\sum_{j=1}^{d}\frac{{\left({\hat{\mu }}_{j}-\mu_{j}^{\mathrm{ref}}\right)}^{2}}{{\left(\sigma_{j}^{\mathrm{ref}}\right)}^{2}}\right\}}^{1/2},$$

where $\mu_{j}^{\mathrm{ref}}$ and $\sigma_{j}^{\mathrm{ref}}$ are the reference mean and standard deviation for component *j*; the table also gives the corresponding maximum and RMS relative SD errors.

The transport-only rows have maximum standardized mean errors near 0.29. Their independence removes serial dependence, so the remaining errors mainly reflect map approximation error, together with finite-sample and reference uncertainty. After residual-slice correction, Gradient CTS and pCN CTS reduce this error to about 0.035 and recover comparable SD summaries. Thus, the same approximate map supplies preconditioning, while residual-slice correction substantially reduces the observed summary error.

### 4.3. Residual-Slice Efficiency Under the Same Preconditioner

Table 5 averages ten independently generated d=20,n=900 target instances; within each instance, the corrected updates share the same preconditioner and residual-slice construction. The Appendix A (Table A1) lists the fixed settings. BlackJAX NUTS ESS/eval is omitted because its counter excludes adaptation, which is included in ESS/sec.

The matched residual-slice comparison is among Gradient CTS, pCN CTS, and the RW hybrid slice update, because all three use the same fixed transport preconditioner and residual-slice target. Within this corrected framework, Gradient CTS reduced the mean IACT from 16.32 to 6.09, while pCN CTS gave a mean IACT of 5.55 and a higher ESS/eval. The remaining rows provide local-gradient, trajectory-based, and random-walk reference points under the same target distribution, but they use different primitive operations and tuning mechanisms. Affine HMC and BlackJAX NUTS attained small IACTs on the default component and radius observables.

Table 6 instead holds one d=20,n=900 target fixed across three chains and reports means and between-chain SDs for vector and energy diagnostics. “Cap” denotes multivariate ESS beyond the 2970 retained draws after batch truncation; E-BFMI and divergences apply only to Hamiltonian methods.

Neither HMC implementation diverged, and BlackJAX NUTS had no maximum-depth hits. Multivariate ESS complements the selected-observable IACT.

### 4.4. Diagnosing the Remaining Mixing Cost

The following diagnostics probe the method-dependent factors $a_{T}$ and $c_{\mathrm{slice}}$ on selected observables; $\gamma_{\mathrm{ref}}$ supplies the reference mixing scale. Hessian probes assess local whitening through (10); movement and feasibility probes assess motion inside transported slices. The Gaussian-probe statistic uses standard-Gaussian starting states and their empirical covariance, so it is a descriptive local-movement summary. It uses paired pre- and post-move states to estimate

$${\hat{c}}_{\mathrm{move}}=\max\left[0,\;\lambda_{\min}\left\{{\left({\hat{G}}_{\mathrm{probe}}+\lambda I\right)}^{-1/2}{\hat{G}}_{\mathrm{move}}{\left({\hat{G}}_{\mathrm{probe}}+\lambda I\right)}^{-1/2}\right\}\right],$$

where

$${\hat{G}}_{\mathrm{move}}={\left(2N_{\mathrm{probe}}\right)}^{-1}\sum_{i=1}^{N_{\mathrm{probe}}}\left(z_{i}-z_{i}^{\prime }\right){\left(z_{i}-z_{i}^{\prime }\right)}^{⊤}.$$

Here, ${\hat{G}}_{\mathrm{probe}}$ is the empirical covariance of the standard-Gaussian starting states, and $z_{i}^{\prime }$ is the state obtained from $z_{i}$ after the selected within-slice update. Feasibility is the fraction of inner proposals satisfying the slice constraint before Metropolis acceptance. Table 7 combines these diagnostics for the two logistic cases and the analytic Gaussian last-layer target. Its “Median Hessian κ” column reports the empirical median transformed-space condition number. Proposition 2 provides the deterministic link between the Hessian-error diagnostic and a regional condition-number bound.

In the d=20 logistic case, the affine preconditioner left moderate Hessian distortion, while Gradient and pCN CTS moved farther than RW hybrid slice. The d=80 case showed weaker whitening and movement. The Gaussian case had an almost identity transformed-space Hessian but limited within-slice movement. Table 8 fixes scales while varying the preconditioner or *m*: Gradient 0.65 in panel (a), and Gradient 0.65 with RW 0.70 in panel (b).

The full Laplace preconditioner gave smaller local distortion and lower IACT than identity or diagonal preconditioning. Increasing *m* raised the Gaussian-probe movement statistic and lowered IACT for both residual-slice updates, while Gradient CTS kept nearly flat ESS/eval because each outer transition became more expensive. At every tested *m*, the gradient-assisted proposal gave more movement and lower IACT than the matched RW hybrid slice update. Table 9 gives three-chain Gradient CTS sensitivity to scale, *m*, and preconditioner; panel (c) selects one scale per preconditioner. Appendix A lists the settings.

Scales 0.50–0.80 gave similar IACT and ESS/sec, whereas scale 1.00 was less efficient. Increasing *m* lowered IACT but left ESS/eval nearly unchanged for m≥2 and reduced ESS/sec relative to m=1. After separate retuning, IACT fell from 130.55 and 59.03 under identity and diagonal maps to 6.78 under the full Laplace map, consistent with the Hessian condition numbers 44.40, 37.94, and 1.51.

### 4.5. Broader Target Settings

The remaining experiments extend the correction-and-diagnosis pattern beyond the main logistic example. The cases serve distinct roles: a higher-dimensional logistic target stresses fixed affine preconditioning in a larger smooth distribution, a Bayesian lasso target introduces a non-smooth density, a banana target studies imperfect nonlinear transport and shape recovery, and a Gaussian reference case provides a calibrated setting in which affine methods have direct access to the target geometry. The first case is an 80-dimensional logistic-regression target with the same fixed Laplace-affine preconditioning. Table 10 compares the corrected updates with affine MALA and HMC on this target.

Table 10 shows the same ordering among corrected methods as in the smaller logistic target. Under the same fixed transport preconditioner and residual-slice correction, Gradient CTS reduced the mean IACT from 109.50 to 26.01, while pCN CTS gave a mean IACT of 24.24 with higher ESS/eval. Affine HMC remained faster on these smooth target-space observables. The diagnostics in Table 7 show less complete local whitening than in the d=20 case, while the Gaussian-probe movement statistic still separates pCN and Gradient CTS from RW hybrid slice.

The target sequence uses d=20,80,200,500 and n=max(400,2d), so dimension, n/d, and predictor saturation vary together. Table 11 gives three-chain Gradient CTS results, and Appendix A.2 gives pCN CTS. IACT near 400 indicates unresolved long correlation.

Full-Laplace preconditioning reduced IACT at d=20 and 80, but mixing remained poor for both maps at d=200 and 500.

Two further cases examine incomplete approximate transports. The non-smooth case is a 40-dimensional Bayesian lasso regression [31]; a smooth surrogate is used to construct its affine preconditioner, while the corrected chains use the original lasso density. The nonlinear case is a ten-dimensional banana target with curvature $b_{\mathrm{tar}}=0.25$ and an imperfect transport using $b_{\mathrm{map}}=0.14$. Table 12 compares the corrected updates with RW hybrid slice and external baselines.

For lasso, pCN and Gradient CTS reduced the mean IACT from 85.24 for matched RW hybrid slice to 13.51 and 17.00, respectively. The subgradient MALA row provides a separate baseline. For the banana target, both corrected methods also improved on matched RW hybrid slice and un-preconditioned RWM.

The banana example adds a shape diagnostic that component-wise summaries alone can miss. Its target curvature is $b_{\mathrm{tar}}=0.25$, and the diagnostic regresses the second component on the centered quadratic feature $x_{1}^{2}-s_{0}^{2}$. Transport-only samples from the imperfect nonlinear transport map can have small component-wise mean error while still estimating this curvature inaccurately. Table 13 presents this shape diagnostic against analytic moments.

**Figure 1:**
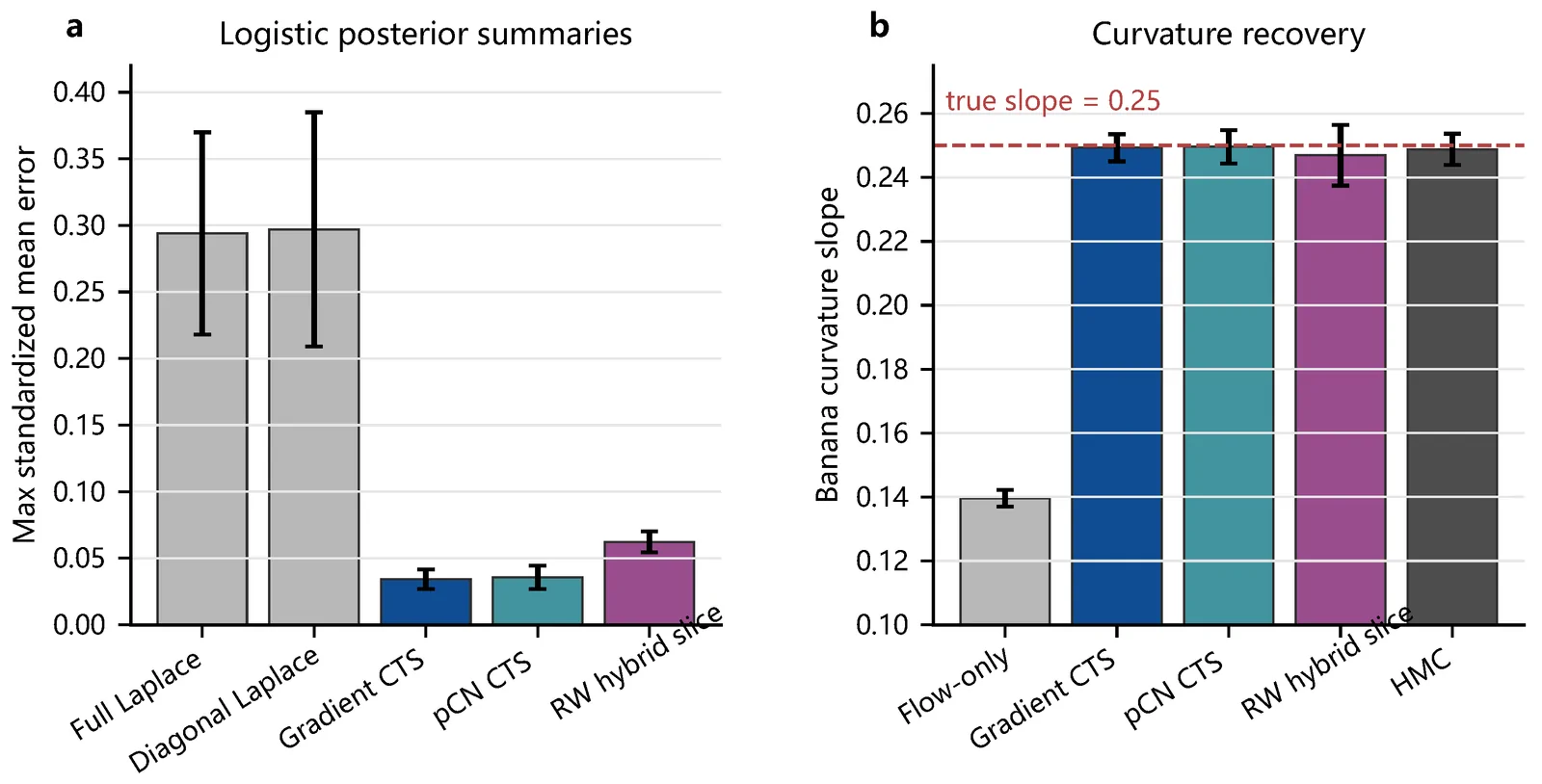
Transport-only bias and corrected recovery. (**a**) Maximum standardized mean error for transport-only samples and corrected chains. (**b**) Curvature-slope recovery under imperfect nonlinear transport. Bars show means over independent runs with one SD.

In the transport-only comparison, the maximum mean error is small, but the curvature slope is about 0.140, compared with the analytic value of 0.25. The contrast shows why the shape diagnostic is needed: component-wise mean errors alone do not reveal this transport-induced curvature bias. When the same nonlinear transport map is used as a fixed transport preconditioner and residual-slice correction is applied, the corrected chains recover slopes near the analytic value. Figure 1 summarizes the logistic summary-recovery and banana curvature-recovery results.

**Figure 2:**
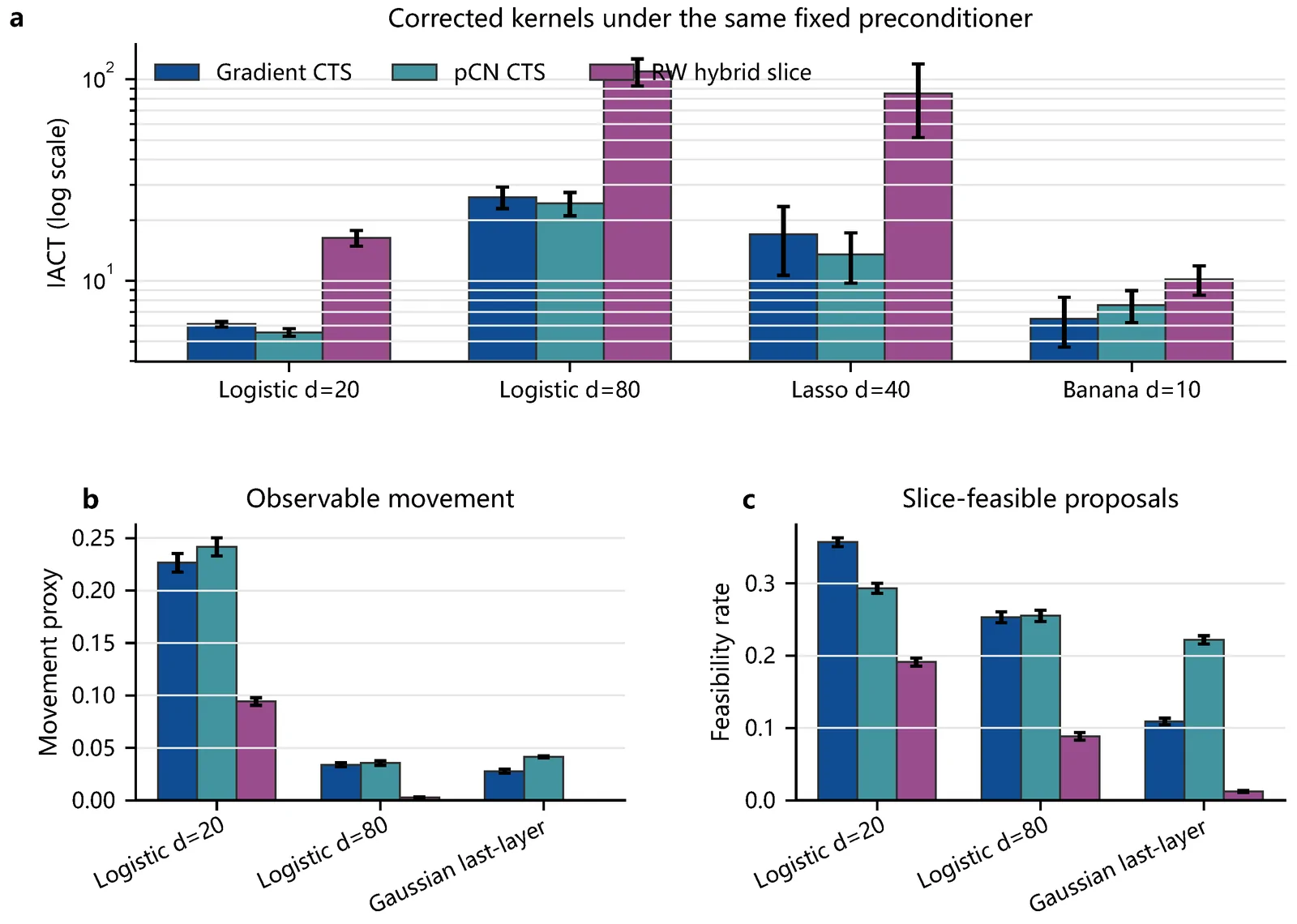
Efficiency and diagnostics under correction. (**a**) IACT comparisons for corrected methods under fixed transport preconditioners. (**b**) Movement-proxy statistics. (**c**) Slice-feasibility rates. Bars show means over independent runs with one SD.

The Gaussian reference case is a setting in which strong affine methods have direct access to the target geometry. For this analytic Gaussian last-layer target, independent sampling and affine HMC had IACTs of 1.02 and 4.16, versus 40.64 and 61.25 for corrected pCN and Gradient CTS. Figure 2 summarizes the efficiency and diagnostic pattern behind the corrected-method comparisons. The non-random-walk updates reduced IACT relative to RW hybrid slice and also had greater movement and feasibility, although their relative ordering varied across diagnostics.

### 4.6. Normalizing-Flow Transport and Corrected Sampling

We evaluated Real NVP checkpoints on a ten-dimensional banana target; the untrained checkpoint was the identity map. Uncorrected flow samples, pCN CTS, and a fixed-flow TESS-style update shared each frozen map [27]. Table 14 reports map quality and corrected efficiency; Appendix A gives the settings. Importance ESS fraction and log-weight SD are map-quality proxies, and TESS bracket evaluations count residual evaluations to the first feasible proposal.

Training raised the importance ESS fraction from 0.0247 to 0.9732 and reduced log-weight SD from 1.346 to 0.186. The projected ${\hat{c}}_{\mathrm{slice}}^{\left(3\right)}$ at steps 0, 100, 500, and 1000 was 0.202, 0.207, 0.240, and 0.232, respectively; like the uncorrected-flow curvature slope, it was non-monotone. Joint map–Jacobian evaluation required 2.03–2.22 ms per batch of 256 states. At the trained checkpoints, pCN CTS acceptance was at least 0.949, and the mean IACT fell from 33.4 to 7.0–9.0. The fixed-flow TESS-style update reduced its bracket cost from 2.60 to about 1.2 residual evaluations and reached IACT 3.4–4.2. Both corrected methods benefited from the trained map, while the TESS-style update was more efficient at these checkpoints.

## 5. Discussion

Residual-slice correction assigns distinct roles to transport geometry, residual correction, and conditional movement. A Laplace-affine map, independent-sample covariance map, or flow-based nonlinear map can improve the geometry explored by a Markov chain. In transformed coordinates, the part of the target not captured by this preconditioner appears as a residual density factor. Residual-slice correction accounts for that factor through an auxiliary-level variable, while a conditional Markov kernel supplies the implementable move within the corresponding residual feasible set. Theorem 2 summarizes this separation through ${\mathrm{gap}}_{D}\left(P_{T}^{\mathrm{hyb},m}\right)\geq c_{\mathrm{slice}}a_{T}\gamma_{\mathrm{ref}}$. The projected diagnostics evaluate selected state summaries, while the constants in the bound are global.

The computational results reflect this decomposition. Transport-only comparisons produced visible summary or shape errors in the diagnostic cases, whereas corrected chains used the same fixed preconditioner and recovered those features. Within the framework, the non-random-walk updates improved on the matched RW hybrid slice baseline in the logistic, lasso, and banana examples. The Gaussian reference case gave the complementary boundary: specialized affine methods were more efficient when the target geometry was available in closed form. For the learned nonlinear map, improved weight diagnostics coincided with lower corrected-chain dependence and lower TESS-style bracket cost. Uncorrected-flow curvature and the projected diagnostic remained non-monotone.

External reference methods such as HMC and NUTS provide trajectory-based scales for the same target distribution but use different moves, costs, and tuning rules. Matched residual-slice comparisons test the correction mechanism under the same invariant conditional distribution. The RW hybrid slice update is the matched baseline because it uses the same fixed transport preconditioner, base–residual split, and invariant slice conditional distribution as the other corrected methods.

Autocorrelation and computational cost capture different aspects of efficiency. Each feasibility attempt repeats the residual evaluation, so increasing *m* can reduce IACT without improving ESS/sec. Learned flows also incur training and map–Jacobian costs. The target density dominated the affine-map calculation in the d=20 example, but this balance depends on the target and implementation. Map quality and conditional movement should therefore be assessed separately.

Full-Laplace preconditioning improved mixing at d=20 and 80, but neither tested map resolved the long correlations at d=200 and 500. Dense maps require $O(d^{2})$ evaluation, and dense generalized-linear-model fitting costs $O(nd^{2}+d^{3})$ per Newton step. Applications with thousands of unknowns call for structured maps and more global updates.

## 6. Conclusions

We developed residual-slice correction as a sampling framework that uses an approximate transport map as a fixed preconditioner. Slice augmentation and invariant conditional movement preserve the target distribution. The Dirichlet-form gap bound separates within-slice movement, transport–reference comparison, and reference mixing. The experiments show recovery of distorted summaries and shape, while the matched comparisons reveal substantial differences among within-slice updates. Practical efficiency still depends on map quality and movement within residual slices. Structured transports and more global updates are natural directions for settings beyond those tested here.

## Figures and Tables

**Table 1:** Proposals and acceptance rules for the three residual-conditional kernels.

Kernel	Proposal	Acceptance Rule	Fixed Parameter
RW hybrid slice	y=z+σξ	(2) with ratio $b_{T}\left(y\right)/b_{T}\left(z\right)$.	σ
Gradient CTS	$y=z+\epsilon^{2}\nabla \log b_{T}\left(z\right)/2+\epsilon \xi$	(2) with the forward–reverse proposal ratio.	ϵ
pCN CTS	$y=\sqrt{1-\beta^{2}}z+\beta \xi /\sqrt{2\tau }$	For a Gaussian base, accept exactly when $y\in S_{T,u}$.	β

**Table 2:** Leading computational cost per retained outer transition.

Method	One-Time Construction	Leading Cost per Outer Transition
RWM	None	$C_{\rho }+O\left(d\right)$
MALA	None	$C_{\nabla \rho }+O\left(d\right)$
HMC	None	$L_{\mathrm{HMC}}\left\{C_{\nabla \rho }+O\left(d\right)\right\}$
NUTS	Warm-up/adaptation	$O\left(2^{D_{\mathrm{NUTS}}}\right)\left\{C_{\nabla \rho }+O\left(d\right)\right\}$
RW hybrid slice	Construct/fix *T*	$m\{C_{T}+C_{J}+C_{\rho }+C_{b}+O\left(d\right)\}$
pCN CTS	Construct/fix *T*	$m\{C_{T}+C_{J}+C_{\rho }+O\left(d\right)\}$
Gradient CTS	Construct/fix *T*	$m\{C_{T}+C_{J}+C_{\rho }+C_{b}+O\left(d\right)\}$
Fixed-flow TESS-style	Train Real NVP	$N_{\mathrm{br}}\left\{C_{T,J}+C_{\rho }+O\left(d\right)\right\}$

**Table 3:** Component timings for the d=20,n=900 logistic target.

Component	Median Time (μs)	Range Across Batches (μs)
Target density	97.1	94.4–102.6
Target density and gradient	200.9	198.3–213.9
Dense affine map	2.25	2.20–2.62
Cached affine log-Jacobian	0.079	0.079–0.080
Residual evaluation, end to end	102.6	101.2–105.6
Feasibility comparison after residual evaluation	0.089	0.089–0.093

**Table 4:** Summary recovery assessment.

Transport Use	Method	Reference	Max. Mean Error	RMS Mean Error	Max./RMS SD Error
Transport-only	Full Laplace	affine HMC	0.294 (0.076)	0.143 (0.027)	0.122 (0.018)/0.063 (0.009)
Transport-only	Diagonal Laplace	affine HMC	0.297 (0.088)	0.142 (0.026)	0.120 (0.015)/0.062 (0.008)
Corrected	Gradient CTS	affine HMC	0.034 (0.007)	0.016 (0.003)	0.089 (0.013)/0.037 (0.003)
Corrected	pCN CTS	affine HMC	0.036 (0.009)	0.015 (0.002)	0.094 (0.021)/0.042 (0.007)
Corrected	RW hybrid slice	affine HMC	0.062 (0.008)	0.029 (0.004)	0.090 (0.012)/0.040 (0.004)
Local-gradient	Affine MALA	affine HMC	0.071 (0.014)	0.034 (0.009)	0.096 (0.020)/0.042 (0.005)

**Table 5:** Logistic-regression sampling comparison.

Method	IACT	ESS/Eval	ESS/Sec	Acceptance
Gradient CTS	6.09 (0.19)	0.0192 (0.0006)	90.98 (3.40)	0.363 (0.002)
pCN CTS	5.55 (0.24)	0.0289 (0.0012)	131.38 (4.37)	0.298 (0.002)
RW hybrid slice	16.32 (1.46)	0.0083 (0.0008)	35.48 (3.48)	0.138 (0.001)
Affine MALA	6.25 (0.23)	0.1281 (0.0047)	441.74 (17.20)	0.745 (0.003)
Affine HMC	1.00 (0.00)	0.0571 (0.0000)	211.98 (5.16)	0.791 (0.038)
BlackJAX NUTS	1.51 (0.33)	–	412.37 (91.72)	0.902 (0.016)
Affine RWM	198.22 (19.81)	0.0041 (0.0004)	14.77 (1.38)	0.666 (0.002)
HMC	53.41 (37.72)	0.0020 (0.0026)	7.85 (9.79)	0.749 (0.093)
MALA	107.06 (27.82)	0.0079 (0.0018)	28.11 (6.08)	0.760 (0.067)
RWM	352.47 (76.11)	0.0024 (0.0005)	8.95 (1.90)	0.237 (0.039)

**Table 6:** Standard diagnostics for the fixed d=20 logistic target.

Method	IACT	Multiv. ESS	ESS/Sec	E-BFMI	Divergences
Gradient CTS	6.48 (0.80)	704 (40)	225.7 (26.4)	–	–
pCN CTS	5.13 (0.38)	742 (23)	295.8 (22.4)	–	–
Affine HMC	1.66 (0.11)	2970 (cap)	164.5 (11.5)	0.930 (0.089)	0
HMC	5.65 (2.39)	2720 (74)	48.8 (17.7)	1.000 (0.042)	0
BlackJAX NUTS	1.90 (0.29)	2970 (cap)	287.1 (28.9)	1.009 (0.038)	0

**Table 7:** Selected-state efficiency diagnostics; Proposition 2 gives the related regional condition-number bound.

Target	Hessian Error (95th perc.)	Median Hessian κ	Gradient Move	pCN Move	RW Move	Gradient Feas.	pCN Feas.	RW Feas.
Logistic	0.492 (0.075)	1.570 (0.083)	0.227 (0.009)	0.242 (0.009)	0.094 (0.004)	0.357 (0.006)	0.293 (0.007)	0.191 (0.006)
Logistic, d=80	1.239 (0.172)	3.136 (0.281)	0.0339 (0.0018)	0.0356 (0.0021)	0.0027 (0.0006)	0.253 (0.007)	0.255 (0.008)	0.089 (0.005)
Gaussian last-layer	$8.8\times 10^{-7}$ ($4.0\times 10^{-7}$)	1.000001 (0.000000)	0.028 (0.002)	0.042 (0.001)	0.000 (0.000)	0.109 (0.005)	0.222 (0.006)	0.013 (0.001)

**Table 8:** Mechanism checks.

(a) Transport-quality ablation for Gradient CTS.						
**Preconditioner**		**IACT**	**ESS/Eval**	**Hessian Error (95th perc.)**	**Hessian** ***κ***	**Gaussian-Probe Move**
Identity		89.29 (35.49)	0.0015 (0.0005)	542.70 (16.67)	47.73 (1.56)	0.004 (0.003)
Diagonal Laplace		56.24 (10.77)	0.0022 (0.0005)	14.87 (0.62)	34.59 (2.50)	0.104 (0.069)
Full Laplace		8.53 (2.44)	0.0155 (0.0039)	0.514 (0.145)	1.576 (0.143)	0.121 (0.023)
(b) Inner-step sensitivity under the full Laplace preconditioner.						
m	**Gradient IACT**	**RW IACT**	**Gradient ESS/Eval**	**RW ESS/Eval**	**Gradient Move**	**RW Move**
1	32.88	87.72	0.0171	0.0073	0.029	0.005
2	16.50	39.76	0.0167	0.0080	0.064	0.016
5	6.45	15.13	0.0171	0.0083	0.162	0.064
10	3.22	8.17	0.0171	0.0077	0.302	0.142

**Table 9:** Gradient CTS sensitivity analysis.

(a) Proposal-scale grid with *m* = 5 and full Laplace transport.				
**Scale**	**Acceptance**	**IACT**	**ESS/Eval**	**ESS/Sec**
0.35	0.628	14.28	0.00725	97.4
0.50	0.487	6.83	0.01573	193.6
0.65	0.366	6.18	0.01927	226.1
0.80	0.252	6.05	0.02119	222.0
1.00	0.141	9.60	0.01543	154.7
(b) Inner-step grid at frozen scale 0.65.				
m	**IACT**	**Multiv. ESS**	**ESS/Eval**	**ESS/Sec**
1	28.84	133	0.02098	236.4
2	17.69	214	0.01684	192.0
5	6.95	480	0.01714	196.3
10	3.65	856	0.01745	200.0
(c) Transport ablation with a separately frozen scale for each map.				
**Transport**	**Scale**	**Acceptance**	**IACT**	**Median Hessian** ***κ***
Identity	0.05	0.297	130.55	44.40
Diagonal Laplace	0.35	0.284	59.03	37.94
Full Laplace	0.80	0.261	6.78	1.51

**Table 10:** d=80 logistic-regression sampling comparison.

Method	IACT	ESS/Eval	ESS/Sec	Acceptance
Gradient CTS	26.01 (3.18)	0.0046 (0.0005)	18.29 (2.11)	0.276 (0.007)
pCN CTS	24.24 (3.22)	0.0063 (0.0008)	23.10 (3.12)	0.280 (0.004)
RW hybrid slice	109.50 (16.80)	0.0013 (0.0002)	4.45 (0.67)	0.057 (0.001)
Affine MALA	96.75 (12.37)	0.0079 (0.0009)	24.08 (2.82)	0.989 (0.001)
Affine HMC	1.03 (0.04)	0.0607 (0.0025)	199.39 (10.78)	0.981 (0.001)

**Table 11:** Logistic-regression target-sequence scaling for Gradient CTS.

*d*	Transport	Scale	Acceptance	IACT	ESS/Sec	Fit Time (s)
20	Diagonal Laplace	0.80	0.230	32.2	243.6	0.003
20	Full Laplace	0.80	0.266	17.4	489.4	0.002
80	Diagonal Laplace	0.50	0.063	269.0	9.5	0.028
80	Full Laplace	0.35	0.264	42.0	49.4	0.138
200	Diagonal Laplace	0.10	0.232	393.7	6.4	0.022
200	Full Laplace	0.10	0.268	387.0	4.3	0.423
500	Diagonal Laplace	0.05	0.166	391.2	4.8	0.041
500	Full Laplace	0.05	0.125	401.4	2.4	0.450

**Table 12:** Non-smooth and nonlinear targets.

Target	Method	IACT	ESS/Eval	Full-Gradient Evals	Acceptance
Lasso	pCN CTS	13.51 (3.79)	0.0126 (0.0030)	0	0.229 (0.008)
Lasso	Gradient CTS	17.00 (6.36)	0.0090 (0.0024)	0	0.144 (0.008)
Lasso	RW hybrid slice	85.24 (33.91)	0.0020 (0.0006)	0	0.025 (0.006)
Lasso	Affine subgradient MALA	23.01 (2.25)	0.0351 (0.0035)	5001	0.949 (0.003)
Lasso	RWM	520.89 (202.24)	0.0018 (0.0008)	0	0.172 (0.010)
Banana	Gradient CTS	6.49 (1.81)	0.0179 (0.0044)	0	0.460 (0.005)
Banana	pCN CTS	7.57 (1.37)	0.0219 (0.0046)	0	0.538 (0.006)
Banana	RW hybrid slice	10.16 (1.70)	0.0119 (0.0019)	0	0.280 (0.002)
Banana	HMC	14.40 (3.46)	0.0048 (0.0009)	60001	0.997 (0.001)
Banana	RWM	102.43 (40.06)	0.0086 (0.0024)	0	0.402 (0.009)

**Table 13:** Banana curvature recovery.

Method	Target-Preserving	Max. Mean Error	Max. SD Error	Slope	Rel. Slope Error
Transport-only	no	0.026 (0.009)	0.239 (0.009)	0.140 (0.003)	0.442 (0.011)
Gradient CTS	yes	0.061 (0.020)	0.046 (0.030)	0.249 (0.004)	0.015 (0.008)
pCN CTS	yes	0.064 (0.023)	0.054 (0.022)	0.250 (0.005)	0.017 (0.011)
RW hybrid slice	yes	0.076 (0.023)	0.075 (0.029)	0.247 (0.009)	0.030 (0.024)
HMC	yes	0.065 (0.024)	0.085 (0.073)	0.249 (0.005)	0.015 (0.012)

**Table 14:** Transport quality and corrected fixed-flow sampling across Real NVP checkpoints.

(a) Transport-quality diagnostics; the uncorrected-flow slope targets the analytic value 0.25.					
**Training Step**		**Cumulative Train (s)**	**Importance ESS Fraction**	**Log-Weight SD**	**Uncorrected-Flow** **Slope**
0		0	0.0247	1.346	−0.003
100		0.83	0.9295	0.249	0.220
500		4.27	0.9672	0.231	0.144
1000		8.98	0.9732	0.186	0.161
(b) Corrected-chain efficiency under the same frozen checkpoints.					
**Step**	**pCN IACT**	**pCN ESS/Sec**	**TESS IACT**	**TESS ESS/Sec**	**TESS Bracket Evals**
0	33.41 (15.19)	6.8 (3.7)	100.17 (102.32)	6.7 (4.7)	2.60 (0.20)
100	8.99 (0.72)	21.1 (1.9)	4.24 (0.78)	186.4 (32.4)	1.23 (0.03)
500	7.01 (0.40)	27.3 (1.5)	3.43 (0.59)	238.5 (35.1)	1.17 (0.01)
1000	8.29 (0.82)	23.4 (2.2)	3.87 (0.70)	220.7 (48.3)	1.15 (0.02)

## Data Availability

The code needed to generate the synthetic datasets and reproduce the numerical experiments is provided as Supplementary Code S1 with this manuscript. No private or third-party restricted dataset is used. The numerical examples use synthetic datasets generated by the supplied code.

## Supplementary Materials

The following supporting information can be downloaded at: https://www.mdpi.com/article/10.3390/e28080899/s1, Code S1: Python code used to generate the synthetic datasets and reproduce the numerical experiments.

## Appendix A. Computational Settings and Supporting Results

### Appendix A.1. Computational Settings

**Controls:** All settings were frozen before retained sampling, and scale-selection draws were excluded.

**Target Settings:** The logistic cases use (d,n,correlation)=(20,900,0.7) and (80,1600,0.6), with prior N(0,9I). Lasso uses (d,n)=(40,300), correlation 0.65, noise SD 0.70, penalty 1, smoothing $10^{-3}$, and ridge $10^{-4}$. Banana uses d=10, $b_{\mathrm{tar}}=0.25$, $b_{\mathrm{map}}=0.14$, and scales (1.4,0.55,0.8,…,0.8).

Table A1 presents the fixed residual-slice settings. The base factor is $b_{T}\left(z\right)=\exp\left(-\tau {∥z∥}^{2}\right)$. Gradient, RW, and pCN entries give ϵ, σ, and β, respectively.

**Table A1:** Residual-slice settings used in the reported experiments.

Experiment Block	Base Parameter	Inner Steps *m*	Fixed Proposal Setting
d=20 logistic	τ=0.175	5	Gradient 0.65; pCN 0.45; RW 0.70.
d=80 logistic	τ=0.175	5	Gradient 0.42; pCN 0.25; RW 0.45.
Lasso and imperfect-map banana targets	τ=0.175	5	Gradient 0.70; pCN 0.35; RW 0.70.
Analytic Gaussian last-layer target	τ=0.175	5	Gradient 0.55; pCN 0.25; RW 0.60.
Mechanism and d=20 sensitivity blocks	τ=0.175	1,2,5,10, as shown	Mechanism: Gradient 0.65, RW 0.70; sensitivity settings are reported in Table 9.
Logistic target-sequence scaling	τ=0.175	3	Gradient scales are reported in Table 11; pCN scales are listed in Appendix A.2.
Real NVP checkpoints	τ=0.5	5	pCN β=0.35, prespecified; the fixed-flow TESS-style comparator used its bracket update.

**Replications and Run Lengths:** Summary recovery uses five 30,000-step runs (8000 burn-in). The d=20 logistic and analytic Gaussian comparisons use ten runs of 62,500 (12,500), the d=80 logistic comparison uses ten runs of 24,000 (6000), and the lasso and banana comparisons use ten runs of 5000 (1000). Mechanism, sensitivity, scaling, standard-diagnostic, and learned-flow results use three runs of 10,000 (2500), 2500 (500), 1200 (200), 3500 (500), and 3000 (500). Timings use nine warmed 500-call batches and 2500 transitions. BlackJAX NUTS uses 2000 warm-up/4000 retained draws in Table 5 and 1500/3000 in Table 6. Scale selection uses separate pre-production runs per candidate (400 transitions in sensitivity analyses; 160 in target-sequence scaling), and the selected settings remain fixed in production.

**Learned Transport and Projected Diagnostics:** Real NVP uses six width-32 coupling layers and reverse Kullback–Leibler (KL) training with Adam. The projected diagnostics follow Algorithm 2 with 100 stationary state–slice pairs per run and checkpoint, exact conditional reference draws, and five pCN steps with β=0.35.

### Appendix A.2. Scaling Results for pCN CTS

The frozen pCN scales for the diagonal and full maps were (0.45,0.60), (0.20,0.10), (0.05,0.05), and (0.02,0.02) at d=20,80,200,500, respectively. Full-Laplace preconditioning reduced the mean IACT from 48.6 to 14.9 at d=20 and from 317.7 to 162.0 at d=80. At d=200, the diagonal and full maps gave IACT 396.2 and 395.3; at d=500, they gave 401.9 and 391.6. Thus, both transports retained unresolved long correlation at the two largest targets, matching the pattern in Table 11.

## Appendix B. Preliminaries for the Proofs

This appendix collects proof tools and technical conventions used after the main text.

**Function Spaces and Spectral Gaps:** For a probability measure ϖ, $L^{2}\left(\varpi \right)$ is the space of square-integrable functions and $L_{0}^{2}\left(\varpi \right)=\left\{f\in L^{2}\left(\varpi \right):\int f\;d\varpi =0\right\}$. Given $f\in L^{2}\left(\varpi \right)$, write

$${∥f∥}_{2,\varpi }={\left(\int f^{2}\;d\varpi \right)}^{1/2},\;{\mathrm{Var}}_{\varpi }\left(f\right)=\int {\left(f-\int f\;d\varpi \right)}^{2}d\varpi ,$$

whenever the integrals are finite. For a reversible Markov kernel *P* with invariant distribution π, Dirichlet-form comparisons are written with

$$E_{P}\left(f,f\right)=\frac{1}{2}\int {\left(f\left(x\right)-f\left(y\right)\right)}^{2}\;J_{P}\left(dx,dy\right),$$

where $J_{P}\left(dx,dy\right)=\pi \left(dx\right)P\left(x,dy\right)$ is the symmetric conductance measure. The Dirichlet-form gap is

$${\mathrm{gap}}_{D}\left(P\right)=\underset{f\in L_{0}^{2}\left(\pi \right),\;f\neq 0}{\inf}\frac{E_{P}\left(f,f\right)}{{\mathrm{Var}}_{\pi }\left(f\right)}.$$

Its absolute $L^{2}\left(\pi \right)$ spectral gap is $\mathrm{gap}\left(P\right)=1-∥P∥$, where $∥P∥$ is the operator norm of $P:L_{0}^{2}\left(\pi \right)\to L_{0}^{2}\left(\pi \right)$. For positive reversible kernels, the two gaps coincide; for general reversible kernels, the statements for ${\mathrm{gap}}_{D}$ apply to lazy versions when an absolute spectral-gap formulation is desired.

**Slice Factors and Level Sets:** Recall that $\tilde{\rho }=b_{T}\omega_{T}$ on Z. The pair is admissible when the residual conditional distributions are proper, as specified in Definition 1, and its level-set function $l_{T}$ is defined in Section 2.

**Proposition A1.** 

*Let $\tilde{\rho }$ be a measurable unnormalized density on Z with $0<\int_{Z}\tilde{\rho }\left(z\right)dz<\infty$ and $0<\tilde{\rho }\left(z\right)<\infty$ on its support.*

(a) *If $b_{T}:Z\to \left(0,\infty \right)$ is measurable, finite, and integrable, then $\omega_{T}\left(z\right)=\tilde{\rho }\left(z\right)/b_{T}\left(z\right)$ gives an admissible pair $\left(b_{T},\omega_{T}\right)$.*
(b) *If $b_{T}\left(z\right)=1$ and $\omega_{T}\left(z\right)=\tilde{\rho }\left(z\right)$, then the pair is admissible whenever every proper superlevel set $\left\{z\in Z:\tilde{\rho }\left(z\right)>u\right\}$ has a finite positive Lebesgue measure.*

The experimental Gaussian bases $b_{T}\left(z\right)=\exp\left(-\tau {∥z∥}^{2}\right)$, τ>0, fall under part (a). Part (b) covers the full-density split $b_{T}=1$, $\omega_{T}=\tilde{\rho }$. The proof is given in Appendix D.

**Transport between the Two Spaces:** The map T:Z→X sends transformed-space variables *z* to original-space variables x=T(z). For every transformed-space Borel set $\tilde{B}$, the transformed target satisfies $\tilde{\pi }\left(\tilde{B}\right)=\pi \left(T\left(\tilde{B}\right)\right)$. If B⊆X is an original-space Borel set, its transformed-space counterpart is $\tilde{B}=T^{-1}\left(B\right)=\left\{z\in Z:T\left(z\right)\in B\right\}$. For a transformed-space kernel $\tilde{P}$, its original-space conjugate is $P_{T}\left(x,B\right)=\tilde{P}\left(T^{-1}\left(x\right),\tilde{B}\right)$. The unitary map associated with the transport is $U:L^{2}\left(\pi \right)\to L^{2}\left(\tilde{\pi }\right)$, (Uf)(z)=f(T(z)).

## Appendix C. Proofs of Main Results

The main results are proven in the order in which they appear in Section 3. To prove Theorem 1 and transfer the comparison bounds to the original space, we use the following transport-conjugacy lemma; its proof is given in Appendix D.

**Lemma A1.** 

*Let $\tilde{P}$ be invariant with respect to $\tilde{\pi }$. Then, the transported kernel $P_{T}$ is invariant with respect to π. If $\tilde{P}$ is reversible with respect to $\tilde{\pi }$, then $P_{T}$ is reversible with respect to π and*

$${\mathrm{gap}}_{D}\left(P_{T}\right)={\mathrm{gap}}_{D}\left(\tilde{P}\right).$$

*The same transport conjugacy also preserves the absolute $L^{2}$ spectral gap whenever that gap is used.*

### Appendix C.1. Proof of Theorem 1

Let

$$Z_{T}=\int_{Z}b_{T}\left(z\right)\omega_{T}\left(z\right)dz,$$

which is positive and finite by Assumption 1 and Definition 1. Define the joint slice measure on transformed states and levels by

$$\eta_{T}\left(dz,du\right)=Z_{T}^{-1}b_{T}\left(z\right)1\left\{0<u<\omega_{T}\left(z\right)\right\}dz\;du.$$

Integrating out *u* gives the *z*-marginal

$$Z_{T}^{-1}\tilde{\rho }\left(z\right)dz=\tilde{\pi }\left(dz\right).$$

Conditional on *z*, the level *u* is uniform on $\left(0,\omega_{T}\left(z\right)\right)$. Conditional on *u*, the density of *z* is proportional to $b_{T}\left(z\right)1\left\{z\in S_{T,u}\right\}$, so the conditional distribution is $\nu_{T,u}$ in (1) for $\eta_{T}^{\left(u\right)}$-almost every proper slice level.

For $\eta_{T}^{\left(u\right)}$-almost every *u*, $K_{T,u}$ is invariant with respect to $\nu_{T,u}$ by Assumption 1. Hence, $K_{T,u}^{m}$ is also invariant with respect to $\nu_{T,u}$ for every m≥1. The transformed hybrid transition first samples *u* from the conditional distribution of *u* given *z* under $\eta_{T}$, and then it applies $K_{T,u}^{m}$ to the *z* component. For bounded measurable *g*,

$$\begin{array}{cc} \int {\tilde{P}}_{T}^{\mathrm{hyb},m}g\left(z\right)\;\tilde{\pi }\left(dz\right) & =\int_{0}^{\infty }\int_{S_{T,u}}K_{T,u}^{m}g\left(z\right)\;\nu_{T,u}\left(dz\right)\;\eta_{T}^{\left(u\right)}\left(du\right)\\ & =\int_{0}^{\infty }\int_{S_{T,u}}g\left(z\right)\;\nu_{T,u}\left(dz\right)\;\eta_{T}^{\left(u\right)}\left(du\right)=\int g\left(z\right)\;\tilde{\pi }\left(dz\right). \end{array}$$

Thus, ${\tilde{P}}_{T}^{\mathrm{hyb},m}$ is invariant with respect to $\tilde{\pi }$. If $K_{T,u}$ is reversible with respect to $\nu_{T,u}$ for $\eta_{T}^{\left(u\right)}$-almost every *u*, then $K_{T,u}^{m}$ is reversible with respect to $\nu_{T,u}$, and for bounded measurable *f* and *g*,

$$\begin{array}{cc} & \int f\left(z\right){\tilde{P}}_{T}^{\mathrm{hyb},m}g\left(z\right)\;\tilde{\pi }\left(dz\right)\\ & \;=\int_{0}^{\infty }\int_{S_{T,u}}f\left(z\right)\;K_{T,u}^{m}g\left(z\right)\;\nu_{T,u}\left(dz\right)\;\eta_{T}^{\left(u\right)}\left(du\right), \end{array}$$

where $\eta_{T}^{\left(u\right)}$ is the *u*-marginal of $\eta_{T}$. Reversibility of $K_{T,u}^{m}$ with respect to $\nu_{T,u}$ makes the last display symmetric in *f* and *g*. Hence, ${\tilde{P}}_{T}^{\mathrm{hyb},m}$ is reversible with respect to $\tilde{\pi }$ in the reversible case.

The original-space kernel $P_{T}^{\mathrm{hyb},m}$ is the conjugate of ${\tilde{P}}_{T}^{\mathrm{hyb},m}$ under the measurable bijection *T*. Lemma A1, applied with $\tilde{P}={\tilde{P}}_{T}^{\mathrm{hyb},m}$, gives invariance and reversibility with respect to π. This completes the proof of Theorem 1. □

### Appendix C.2. Proof of Proposition 1

This proof works in the transformed space. Let $f\in L_{0}^{2}\left(\tilde{\pi }\right)$. We use the same joint slice measure $\eta_{T}$ as in the proof of Theorem 1, and we write $\eta_{T}^{\left(u\right)}$ for its level marginal distribution. Conditional on a slice level *u*, the state distribution is $\nu_{T,u}$. Since

$$\int_{0}^{\infty }\int f{\left(z\right)}^{2}\;\nu_{T,u}\left(dz\right)\;\eta_{T}^{\left(u\right)}\left(du\right)=\int f{\left(z\right)}^{2}\;\tilde{\pi }\left(dz\right)<\infty ,$$

the slice restrictions of *f* are square-integrable for $\eta_{T}^{\left(u\right)}$-almost every *u*.

First, consider the hybrid transition. By construction, one step of ${\tilde{P}}_{T}^{\mathrm{hyb},m}$ draws *u* from the conditional distribution of the level variable and then applies $K_{T,u}^{m}$. Since it is reversible, its Dirichlet form is

$$E_{{\tilde{P}}_{T}^{\mathrm{hyb},m}}\left(f,f\right)=\frac{1}{2}\int {\left(f\left(z\right)-f\left(y\right)\right)}^{2}\tilde{\pi }\left(dz\right){\tilde{P}}_{T}^{\mathrm{hyb},m}\left(z,dy\right).$$

Disintegrating with respect to the slice level gives

(A1) $$E_{{\tilde{P}}_{T}^{\mathrm{hyb},m}}\left(f,f\right)=\int_{0}^{\infty }E_{K_{T,u}^{m}}^{\left(u\right)}\left(f,f\right)\eta_{T}^{\left(u\right)}\left(du\right).$$

In this display, *f* is restricted to the slice when viewed as an element of $L^{2}\left(\nu_{T,u}\right)$. Applying the comparison induced by (3) to (A1) yields

(A2) $$E_{{\tilde{P}}_{T}^{\mathrm{hyb},m}}\left(f,f\right)\geq c_{\mathrm{slice}}\int_{0}^{\infty }{\mathrm{Var}}_{\nu_{T,u}}\left(f\right)\eta_{T}^{\left(u\right)}\left(du\right).$$

Now, consider the ideal transformed slice transition ${\tilde{P}}_{T}^{\mathrm{ideal}}$. Given the same level *u*, it draws the new transformed state independently from $\nu_{T,u}$. Therefore,

$$\begin{array}{cc} E_{{\tilde{P}}_{T}^{\mathrm{ideal}}}\left(f,f\right) & =\frac{1}{2}\int_{0}^{\infty }\;\int \int \;{\left(f\left(z\right)-f\left(y\right)\right)}^{2}\nu_{T,u}\left(dz\right)\nu_{T,u}\left(dy\right)\eta_{T}^{\left(u\right)}\left(du\right)\\ & =\int_{0}^{\infty }{\mathrm{Var}}_{\nu_{T,u}}\left(f\right)\eta_{T}^{\left(u\right)}\left(du\right). \end{array}$$

Combining this identity with (A2) gives the Dirichlet-form comparison

(A3) $$E_{{\tilde{P}}_{T}^{\mathrm{hyb},m}}\left(f,f\right)\geq c_{\mathrm{slice}}E_{{\tilde{P}}_{T}^{\mathrm{ideal}}}\left(f,f\right),\;f\in L_{0}^{2}\left(\tilde{\pi }\right).$$

Because both transformed kernels have invariant distribution $\tilde{\pi }$, their variances are computed with respect to the same measure. Taking the infimum in (A3) after division by ${\mathrm{Var}}_{\tilde{\pi }}\left(f\right)$ gives

$${\mathrm{gap}}_{D}\left({\tilde{P}}_{T}^{\mathrm{hyb},m}\right)\geq c_{\mathrm{slice}}{\mathrm{gap}}_{D}\left({\tilde{P}}_{T}^{\mathrm{ideal}}\right).$$

The last step is to transfer the result to the original space. By Lemma A1, the fixed transport conjugacy preserves the Dirichlet-form gap:

$${\mathrm{gap}}_{D}\left(P_{T}^{\mathrm{hyb},m}\right)={\mathrm{gap}}_{D}\left({\tilde{P}}_{T}^{\mathrm{hyb},m}\right),\;{\mathrm{gap}}_{D}\left(P_{T}^{\mathrm{ideal}}\right)={\mathrm{gap}}_{D}\left({\tilde{P}}_{T}^{\mathrm{ideal}}\right).$$

Substituting these identities into the transformed-space bound gives the stated original-space inequality. This completes the proof of Proposition 1. □

### Appendix C.3. Proof of Theorem 2

The proof again starts in the transformed space. Proposition 1 established the Dirichlet-form comparison

$$E_{{\tilde{P}}_{T}^{\mathrm{hyb},m}}\left(f,f\right)\geq c_{\mathrm{slice}}\;E_{{\tilde{P}}_{T}^{\mathrm{ideal}}}\left(f,f\right),\;f\in L_{0}^{2}\left(\tilde{\pi }\right),$$

which is (A3). The definition of $a_{T}$ gives the transport comparison (6). Combining the two comparisons gives

$$E_{{\tilde{P}}_{T}^{\mathrm{hyb},m}}\left(f,f\right)\geq c_{\mathrm{slice}}a_{T}E_{Q_{T}}\left(f,f\right),\;f\in L_{0}^{2}\left(\tilde{\pi }\right).$$

By the definition $\gamma_{\mathrm{ref}}={\mathrm{gap}}_{D}\left(Q_{T}\right)$,

$$E_{Q_{T}}\left(f,f\right)\geq \gamma_{\mathrm{ref}}{\mathrm{Var}}_{\tilde{\pi }}\left(f\right).$$

Therefore,

$$E_{{\tilde{P}}_{T}^{\mathrm{hyb},m}}\left(f,f\right)\geq c_{\mathrm{slice}}a_{T}\gamma_{\mathrm{ref}}{\mathrm{Var}}_{\tilde{\pi }}\left(f\right).$$

Taking the infimum over nonzero $f\in L_{0}^{2}\left(\tilde{\pi }\right)$ yields

$${\mathrm{gap}}_{D}\left({\tilde{P}}_{T}^{\mathrm{hyb},m}\right)\geq c_{\mathrm{slice}}a_{T}\gamma_{\mathrm{ref}}.$$

Finally, Lemma A1 transfers the same Dirichlet-form gap to the original-space kernel $P_{T}^{\mathrm{hyb},m}$. This completes the proof of Theorem 2. □

### Appendix C.4. Proof of Theorem 3

Let $f=\sum_{i=1}^{r}c_{i}\psi_{i}=c^{⊤}\psi$. By bilinearity of the Dirichlet form and covariance,

$$\int E_{K_{T,u}^{m}}^{\left(u\right)}\left(f,f\right)\eta_{T}^{\left(u\right)}\left(du\right)=c^{⊤}G_{\mathrm{hyb}}c,\;\int {\mathrm{Var}}_{\nu_{T,u}}\left(f\right)\eta_{T}^{\left(u\right)}\left(du\right)=c^{⊤}G_{\mathrm{ideal}}c.$$

Because $G_{\mathrm{ideal}}$ is positive definite on $V_{\psi }$, the Rayleigh quotient

$$\frac{c^{⊤}G_{\mathrm{hyb}}c}{c^{⊤}G_{\mathrm{ideal}}c},\;c\neq 0,$$

has minimum

$$\lambda_{\min}\left(G_{\mathrm{ideal}}^{-1/2}G_{\mathrm{hyb}}G_{\mathrm{ideal}}^{-1/2}\right)=c_{\mathrm{slice}}^{\left(r\right)}.$$

Equivalently,

$$c^{⊤}G_{\mathrm{hyb}}c\geq c_{\mathrm{slice}}^{\left(r\right)}c^{⊤}G_{\mathrm{ideal}}c$$

for every vector *c*, and equality is attained along a generalized eigenvector. Hence, no larger constant can satisfy the same inequality on all of $V_{\psi }$. This proves the projected version of (4). The argument for $a_{T}^{\left(r\right)}$ is identical after replacing the numerator matrix $G_{\mathrm{hyb}}$ by the ideal movement matrix $G_{\mathrm{ideal}}$ and the denominator matrix $G_{\mathrm{ideal}}$ by $G_{Q}\left(i,j\right)=E_{Q_{T}}\left(\psi_{i},\psi_{j}\right)$. This completes the proof of Theorem 3. □

### Appendix C.5. Proof of Propositions 2 and 3

For Proposition 2, the additive log-determinant term in ${\tilde{U}}_{L}$ is constant; hence,

$$\nabla^{2}{\tilde{U}}_{L}\left(z\right)=H^{-1/2}\nabla^{2}U\left(T_{L}\left(z\right)\right)H^{-1/2}.$$

The assumption says that every eigenvalue of this matrix lies in the interval

$$\left[1-\varepsilon_{R},\;1+\varepsilon_{R}\right]$$

for $z\in E_{R}$. Thus, for all $v\in R^{d}$ and all $z\in E_{R}$,

$$\left(1-\varepsilon_{R}\right){∥v∥}^{2}\leq v^{⊤}\nabla^{2}{\tilde{U}}_{L}\left(z\right)v\leq \left(1+\varepsilon_{R}\right){∥v∥}^{2}.$$

The inequalities give the local strong-convexity and smoothness statement on $E_{R}$, and the condition-number bound follows by dividing the upper curvature bound by the lower one. This proves Proposition 2.

For Proposition 3, the translation ${\hat{\mu }}_{N}$ does not affect covariance. Let

$$C_{N}=\sum^{-1/2}{\hat{\sum }}_{N}\sum^{-1/2}.$$

The event in the proposition implies

$$\left(1-\varepsilon \right)I⪯C_{N}⪯\left(1+\varepsilon \right)I.$$

The covariance of $z=T_{N}^{-1}\left(x\right)$ is

$$\mathrm{Cov}\left(z\right)={\hat{\sum }}_{N}^{-1/2}\sum {\hat{\sum }}_{N}^{-1/2}.$$

This matrix is similar to ${\hat{\sum }}_{N}^{-1}\sum$. The matrix $C_{N}^{-1}=\sum^{1/2}{\hat{\sum }}_{N}^{-1}\sum^{1/2}$ is also similar to ${\hat{\sum }}_{N}^{-1}\sum$, so Cov(z) and $C_{N}^{-1}$ have the same eigenvalues. Therefore, the eigenvalues of Cov(z) lie between ${\left(1+\varepsilon \right)}^{-1}$ and ${\left(1-\varepsilon \right)}^{-1}$. Hence,

$$\kappa \left\{\mathrm{Cov}\left(z\right)\right\}\leq \frac{{\left(1-\varepsilon \right)}^{-1}}{{\left(1+\varepsilon \right)}^{-1}}=\frac{1+\varepsilon }{1-\varepsilon }.$$

This proves the deterministic covariance-whitening diagnostic. Together with the first part, this completes the proof of Propositions 2 and 3. □

## Appendix D. Proofs of Auxiliary Results

### Appendix D.1. Proof of Proposition A1

For part (a), $\omega_{T}=\tilde{\rho }/b_{T}$, so $b_{T}\omega_{T}=\tilde{\rho }$, and

$$0<\int_{Z}b_{T}\left(z\right)\omega_{T}\left(z\right)dz=\int_{Z}\tilde{\rho }\left(z\right)dz<\infty .$$

Moreover, $\omega_{T}$ is positive and finite on the support of $\tilde{\rho }$ because $b_{T}$ is positive and finite. For every level u>0 with $\tilde{\pi }\left\{\tilde{\rho }/b_{T}>u\right\}>0$,

$$l_{T}\left(u\right)=\int_{Z}b_{T}\left(z\right)1\left\{\tilde{\rho }\left(z\right)/b_{T}\left(z\right)>u\right\}dz\leq \int_{Z}b_{T}\left(z\right)dz<\infty .$$

The set $\left\{\tilde{\rho }/b_{T}>u\right\}$ has a positive Lebesgue measure on the support of $\tilde{\rho }$. If it had zero Lebesgue measure, then $\int_{\left\{\tilde{\rho }/b_{T}>u\right\}}\tilde{\rho }\left(z\right)dz=0$, contradicting $\tilde{\pi }\left\{\tilde{\rho }/b_{T}>u\right\}>0$. Since $b_{T}$ is strictly positive and finite, $l_{T}\left(u\right)>0$. Hence, Definition 1 holds.

For part (b), $b_{T}=1$ and $\omega_{T}=\tilde{\rho }$. The normalization condition is the integrability of $\tilde{\rho }$. The level-set function is $l_{T}\left(u\right)=\lambda_{d}\left\{z\in Z:\tilde{\rho }\left(z\right)>u\right\}$, where $\lambda_{d}$ denotes the *d*-dimensional Lebesgue measure. It is finite and positive by assumption for every proper level. This completes the proof of Proposition A1. □

### Appendix D.2. Proof of Lemma A1

For any original-space Borel set *B*, set $\tilde{B}=T^{-1}\left(B\right)$. Invariance follows from

$$\int P_{T}\left(x,B\right)\;\pi \left(dx\right)=\int \tilde{P}\left(z,\tilde{B}\right)\;\tilde{\pi }\left(dz\right)=\tilde{\pi }\left(\tilde{B}\right)=\pi \left(B\right).$$

For reversibility, let $B_{1},B_{2}$ be original-space Borel sets and set ${\tilde{B}}_{i}=T^{-1}\left(B_{i}\right)$ for i=1,2. Then,

$$\int_{B_{1}}P_{T}\left(x,B_{2}\right)\;\pi \left(dx\right)=\int_{{\tilde{B}}_{1}}\tilde{P}\left(z,{\tilde{B}}_{2}\right)\;\tilde{\pi }\left(dz\right)=\int_{{\tilde{B}}_{2}}\tilde{P}\left(w,{\tilde{B}}_{1}\right)\;\tilde{\pi }\left(dw\right)=\int_{B_{2}}P_{T}\left(y,B_{1}\right)\;\pi \left(dy\right).$$

For the gap identity, the map $U:L^{2}\left(\pi \right)\to L^{2}\left(\tilde{\pi }\right)$, (Uf)(z)=f(T(z)), is unitary because $z∼\tilde{\pi }$ exactly when T(z)∼π. It maps $L_{0}^{2}\left(\pi \right)$ onto $L_{0}^{2}\left(\tilde{\pi }\right)$, and the definition of $P_{T}$ gives $UP_{T}=\tilde{P}U$. Thus, $P_{T}=U^{-1}\tilde{P}U$ on the mean-zero spaces. This unitary equivalence preserves variances and Dirichlet forms, giving ${\mathrm{gap}}_{D}\left(P_{T}\right)={\mathrm{gap}}_{D}\left(\tilde{P}\right)$. It also preserves operator norms and, hence, preserves the absolute $L^{2}$ spectral gap when that gap is used. This completes the proof of Lemma A1. □
